# Landscape of somatic allelic imbalances and copy number alterations in HER2-amplified breast cancer

**DOI:** 10.1186/bcr3075

**Published:** 2011-12-14

**Authors:** Johan Staaf, Göran Jönsson, Markus Ringnér, Bo Baldetorp, Åke Borg

**Affiliations:** 1 Department of Oncology, Clinical Sciences, Lund University and Skåne University Hospital, Barngatan 2B, SE 22185 Lund, Sweden; 2 CREATE Health Strategic Center for Translational Cancer Research, Lund University, BMC C13, SE 22184, Lund, Sweden; 3 Lund Strategic Research Center for Stem Cell Biology and Cell Therapy, Klinikgatan 26, Biomedical Center, Lund University, SE-22184 Lund, Sweden

## Abstract

**Introduction:**

Human epidermal growth factor receptor 2 (HER2)-amplified breast cancer represents a clinically well-defined subgroup due to availability of targeted treatment. However, HER2-amplified tumors have been shown to be heterogeneous at the genomic level by genome-wide microarray analyses, pointing towards a need of further investigations for identification of recurrent copy number alterations and delineation of patterns of allelic imbalance.

**Methods:**

High-density whole genome array-based comparative genomic hybridization (aCGH) and single nucleotide polymorphism (SNP) array data from 260 HER2-amplified breast tumors or cell lines, and 346 HER2-negative breast cancers with molecular subtype information were assembled from different repositories. Copy number alteration (CNA), loss-of-heterozygosity (LOH), copy number neutral allelic imbalance (CNN-AI), subclonal CNA and patterns of tumor DNA ploidy were analyzed using bioinformatical methods such as genomic identification of significant targets in cancer (GISTIC) and genome alteration print (GAP). The patterns of tumor ploidy were confirmed in 338 unrelated breast cancers analyzed by DNA flow cytometry with concurrent BAC aCGH and gene expression data.

**Results:**

A core set of 36 genomic regions commonly affected by copy number gain or loss was identified by integrating results with a previous study, together comprising > 400 HER2-amplified tumors. While CNN-AI frequency appeared evenly distributed over chromosomes in HER2-amplified tumors, not targeting specific regions and often < 20% in frequency, the occurrence of LOH was strongly associated with regions of copy number loss. HER2-amplified and HER2-negative tumors stratified by molecular subtypes displayed different patterns of LOH and CNN-AI, with basal-like tumors showing highest frequencies followed by HER2-amplified and luminal B cases. Tumor aneuploidy was strongly associated with increasing levels of LOH, CNN-AI, CNAs and occurrence of subclonal copy number events, irrespective of subtype. Finally, SNP data from individual tumors indicated that genomic amplification in general appears as monoallelic, that is, it preferentially targets one parental chromosome in HER2-amplified tumors.

**Conclusions:**

We have delineated the genomic landscape of CNAs, amplifications, LOH, and CNN-AI in HER2-amplified breast cancer, but also demonstrated a strong association between different types of genomic aberrations and tumor aneuploidy irrespective of molecular subtype.

## Introduction

Breast cancer (BC) constitutes a heterogeneous group of lesions with differences in clinical presentation, pathological features and biological behavior. Amplification and overexpression of the human epidermal growth factor receptor 2 (*HER2) *(HER2/*neu, ERBB2*) oncogene occur in 15 to 25% of invasive BC [1,2] and define a clinically important subgroup (HER2+). Patients with HER2+ BC have traditionally been associated with poor prognosis [1,3]; however, the advent of HER2-targeted therapies has changed the natural course of the disease for many patients, representing one of the success stories of modern oncology. Unfortunately, not all patients with HER2+ disease benefit from targeted treatment, and some develop treatment resistance over time. It has become evident through microarray-based studies that BC with genomic amplification of *HER2 *(HER2-amplified) constitutes a biologically heterogeneous subgroup of tumors regarding both gene expression patterns and copy number alterations (CNAs) [4,5]. Such genomic profiles have predominantly been obtained from array comparative genomic hybridization (aCGH) [5-7], but more recently single nucleotide polymorphism (SNP) microarrays have become increasingly used, allowing simultaneous detection of both CNAs and allelic imbalance (AI) [8-11]. However, due to disease and data complexity, CNA information has so far mostly been extracted from SNP array data and only recently have robust analysis methods emerged capable of detecting and integrating CNAs and AI [10,12-14]. Consequently, HER2-amplified BC has not yet been thoroughly investigated in this respect. We, therefore, analyzed assembled BC data from different repositories and by integrating these results with our previous study comprising 200 HER2-amplified tumors [5], we were able to define a core set of significant CNAs and recurrent amplifications. Furthermore, using a combination of bioinformatical methods for SNP arrays and quantitative DNA flow cytometry (FCM) we delineated the patterns of loss of heterozygosity (LOH), copy number neutral allelic imbalance (CNN-AI), tumor ploidy, tumor subclonality and occurrence of monoallelic gene amplification. Data from HER2-amplified tumors were compared to data from other subgroups of BC, shedding light on a complex landscape of genomic alterations in a clinically important disease entity.

## Materials and methods

### Tumor material

DNA from 26 frozen tumors with *HER2 *gene amplification was extracted as previously described [5]. SNP analysis was performed at the SCIBLU Genomics Resource Center [15] using the Illumina (Illumina, San Diego, CA, USA) Omni 1 M and 2.5 M Quad beadchips (*n *= 5 and 21, respectively) according to manufacturer's instructions. The 26 cases are referred to as the Lund-HER2-SNP set and are part of a previous study of HER2-amplified BC that was approved by the regional Ethical Committee in Lund (reg. no. LU240-01 and 2009/658), waiving the requirement for informed consent for the study [5]. In addition, genomic profiles were obtained from public repositories for 12 studies [8-11,16-23] comprising 218 primary tumors with amplification of *HER2*, and 16 reported HER2-amplified BC cell lines analyzed by Agilent (Agilent, Santa Clara, CA, USA) aCGH, Illumina SNP beadchips or Affymetrix (Affymetrix, Santa Clara, CA, USA) SNP arrays **[**. HER2-amplified tumor cases were identified based on genomic profiles and displayed marked elevation of *HER2 *copy number (CN) estimates compared to remaining samples from respective data set (Figure S1, Additional file 1). Additionally, 115 of the 218 tumors had public annotations regarding HER2-status, and of these 98% (113) were annotated as HER2+. Gene expression profiles were available for 99 of 218 tumor cases. Gene expression subtypes were assigned based on correlation to PAM50 [24] gene expression centroids obtained from Weigelt *et al*. [25] as further described (Additional file 2 and [26]). Correlation of gene expression data with genomic alterations was performed as described ([27] and Additional file 2). Analysis of differential gene expression for genes in recurrent amplifications was performed as described (Additional file 2). Patient and tumor characteristics are summarized in Table 1 and Table S1 in Additional file 1. SNP data for the Lund-HER2-SNP set are available as series GSE31645 from Gene Expression Omnibus [28].

**Table 1:** Characteristics of individual data sets used in the present study stratified by microarray platform

Data Set	# HER2-amplified	Platform	BAF^A^	GISTIC/GAP ^B^	# Cell lines	Matching GEX	ER: -/+	LN ^C^: -/+	Grade: 1/2/3	PR ^D^: -/+
**GSE10099 **[11]	27	Affymetrix 100K	Yes	Yes/Yes	0	27	11/16	25/0	0/1/10	11/12
**GSE16619 **[9]	11	Affymetrix 250K Nsp	Yes	Yes/Yes	0	NA	2/3	3/8	1/3/7	4/1
	14	Affymetrix 5.0	No	Yes/No	0	NA	5/5	3/9	0/3/11	5/4
**Nikolsky **[16]	9	Affymetrix 250K Sty	Yes	Yes/Yes	0	NA	NA	NA	NA	NA
**GSE19399 **[17]*	32	Affymetrix 250K Sty	Yes	Yes/Yes	6	NA	NA	NA	NA	NA
**GSE7545 **[8]	5	Affymetrix 250K Nsp	Yes	Yes/Yes	0	NA	1/0	0/5	NA	1/0
**GSE13696 **[22]	6	Affymetrix 100K	Yes	No/No	6	NA	NA	NA	NA	NA
**GSK **[23]	4	Affymetrix 250K Nsp	Yes	No/No	4	NA	NA	NA	NA	NA
**Van Loo **[10]	20	Illumina 109K	Yes	Yes/Yes	0	20	14/6	5/14	0/7/13	NA
**GSE11977 **[21]	1	Illumina 550K	Yes	Yes/Yes	0	NA	0/1	1/0	NA	0/1
**GSE20393 **[19]	42	Agilent 244K	No	Yes/No	0	NA	2/1	NA	NA	2/0
**GSE20394 **[18]	15	Agilent 244K	No	Yes/No	0	7	9/6	5/10	0/6/9	4/11
**GSE17907 **[20]	48	Agilent 244K	No	Yes/No	0	45	20/21	12/27	3/10/28	23/18
**Lund-HER2-SNP set**	5	Illumina 1M	Yes	No/No	0	NA	4/1	1/4	NA	4/1
	21	Illumina 2.5M	Yes	No/No	0	NA	15/6	13/8	NA	16/5

^A ^BAF, B Allele Frequency. ^B ^Included in GISTIC analysis and/or GAP analysis. ^C ^LN, Lymph node. ^D ^PR, Progesterone receptor.* Cell lines excluded from GISTIC/GAP analysis.

### SNP and aCGH preprocessing

Affymetrix SNP array data for 108 HER2-amplified tumors or cell lines were obtained from public repositories [17,28]. CEL files were normalized using CRMAv2 [29] and ACNE [30] for generation of CN and B allele frequency (BAF) estimates as further described (Additional file 2). CN and BAF estimates for 47 HER2-amplified cases analyzed by Illumina SNP beadchips were generated using GenomeStudio (Illumina). Illumina 2.5M SNP BAF data were quantile normalized [21] prior to further analysis. Normalized Agilent 244K data were obtained for 105 HER2-amplified cases from Gene Expression Omnibus [28]. Genomic profiles from all array platforms except the Illumina 2.5 M platform and GSE17907 were partitioned using Gain and Loss Analysis of DNA (GLAD) [31]. The Illumina 2.5 M platform was partitioned using Circular Binary Segmentation with α = 0.001 [32], while already partitioned data for GSE17907 were obtained from Gene Expression Omnibus. All genomic profiles were centralized similarly as described [18]. Probe annotations for all array platforms were updated to the hg18 genome build [33]. Partitioned genomic profiles from different array platforms were merged to a common 10,000 bp probe set using a modified version of the method described by Gunnarsson *et al*. [34] (Additional file 2). Data processing steps are further described in Additional file 2.

### Identification of AI and significant CNAs

A modified version of the BAF segmentation [12] software was used to partition BAF estimates from SNP arrays. Partitioned BAF estimates were subsequently integrated with CN data as described (Additional file 2). Genomic Identification of Significant Targets in Cancer (GISTIC) [35] analysis was used to identify significant CNAs using a residual q-value cut-off of 0.05 (Additional file 2). Only data from the 218 primary tumors obtained from public repositories were used in GISTIC analysis. Thus, HER2-amplified cell lines and the Lund-HER2-set were excluded from GISTIC analysis, the latter due to overlap with our previous study [5]. Recurrent high-level amplifications, excluding 17q, were defined as the minimal overlap of amplifications with log2ratio > 1 occurring in ≥ 3% of the 218 samples. Recurrent high-level amplifications on 17q were defined from GISTIC regions of gain on 17q due to the high genomic complexity on this chromosome arm. The fraction of the genome altered by CNAs (CN-FGA) was defined as previously described [5].

### HER2-negative breast cancer SNP data set

A HER2-negative BC reference SNP data set comprising 346 tumors was created by excluding HER2-amplified cases from the Van Loo [10] and GSE10099 [11] data sets. SNP array data were processed similarly as for HER2-amplified cases from respective data set. Gene expression data were available for all 346 cases, and samples were classified according to PAM50 gene expression subtypes (Table S1 in Additional file 1). Unclassified samples, and the few HER2-negative samples classified as belonging to the HER2-enriched molecular subtype in the reference data set were excluded from further analysis, leaving a final number of 308 cases used in subsequent comparisons. The 308 cases were classified as basal-like (*n *= 96), luminal A (*n *= 88), luminal B (*n *= 90), and normal-like (*n *= 34).

### GAP analysis

Integrated AI and CN data for primary tumors analyzed by SNP arrays (99 HER2-amplified, 308 HER2-negative) merged to the 10,000 bp probe set were subjected to Genome Alteration Print (GAP) [13] analysis for estimation of allele-specific copy numbers, fraction of aberrant cells and an *in silico *tumor ploidy (referred to as GAP-ploidy hereon). Frequency of gains and losses from GAP profiles, relative to the GAP-ploidy, as well frequency of CNN-AI were calculated as described [10]. LOH frequency was defined as described [10] with the addition that LOH was not inferred for SNPs called as gain. The fraction of the genome altered by LOH (LOH-FGA) and CNN-AI (CNN-FGA) were defined similarly as for CNAs.

### Identification of subclonal CN loss events

To estimate the frequency of subclonal CN events in HER2-amplified and HER2-negative tumors, only regions identified as CN loss relative to the GAP-ploidy were considered for further analysis. For each sample, a set of CN loss regions were identified by selecting loss regions for which the total GAP CN differed by ≥ 0.6 from another CN loss region. This set of CN loss regions were filtered so that only regions with: 1) GLAD partitioned CN log2ratio < -.12, and 2) size > 100 markers remained. Finally, to reduce influence of small shifts in the data an absolute difference > 0.2 in GLAD partitioned log2ratios between the CN loss regions in the set with the highest and lowest GAP CNs, respectively, were required for the sample to be annotated as harboring subclonal CN loss.

### DNA flow cytometry analysis

DNA FCM data for a cohort of 338 BC patients with available BAC aCGH and gene expression data (assembled from [5,36] and unpublished data) were obtained as previously described [37]. DNA histograms were evaluated using ModFitLT (Verity Software House, Topsham, ME, USA) as described [38]. An experimental tumor ploidy (FCM-ploidy) was calculated as the sum of each detected peak's DNA-index times the fraction of cells present in the peak, representing a measurement of the total amount of DNA in analyzed samples. Tissues from diploid tumor cases were verified by touch imprinting and subsequent HE staining to contain cancer cells. PAM50 molecular classifications and BAC aCGH data were available for 282 and 234 cases respectively. Patient and tumor characteristics for FCM analyzed cases are summarized in Table S1 in Additional file 1.

## Results

### Repeated observations of significant CNAs and recurrent amplifications in HER2-amplified breast cancer

**Figure 1:**
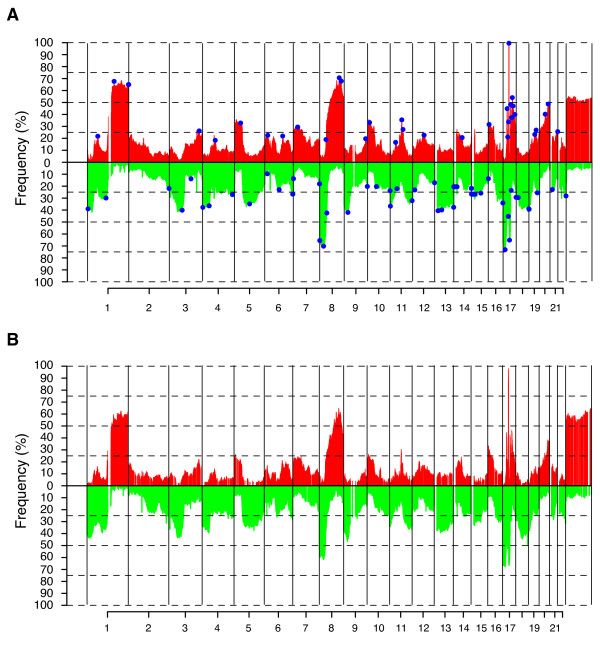
**Pattern of CNAs in HER2-amplified breast cancer**. (**A**) Frequency of copy number gain (red) and loss (green) across 218 HER2-amplified tumors using log2ratio ± 0.12 as thresholds for CN gain and loss respectively. Blue regions indicate significant CNAs identified by GISTIC analysis. (**B**) Frequency of copy number gain (red) and loss (green) across 99 HER2-amplified tumors analyzed by SNP microarrays. Copy number gain and loss were called relative to the GAP-ploidy as described [10].

GISTIC analysis of 218 primary HER2-amplified tumors profiled by high-density SNP or oligonucleotide aCGH identified 80 regions (34 gains and 46 losses) distributed across all autosomes (Figure 1A, Additional file 3). Of the 80 GISTIC regions, 17 overlapped with GISTIC regions identified in our previous study of 200 HER2-amplified BCs profiled by BAC aCGH [5] using the same q-value threshold (Additional file 4). Moreover, an additional 19 GISTIC regions were located in close vicinity (< 2 Mbp) from previously identified GISTIC regions [5] (Additional file 4 and Figure S2A in Additional file 5). Taken together this highlights a core set of genomic regions commonly affected by CNAs in HER2-amplified BC. Integration of gene expression data with CN estimates for genes in these core regions identified numerous genes with significant correlation between mRNA levels and CN estimates, of which some overlapped with findings from our previous BAC study (Additional file 4). The overall pattern of CNAs in HER2-amplified BC was mimicked in 16 HER2-amplified BC cell lines, although with slightly higher prevalence in cell lines, and with a few discrepancies, for example, more frequent losses on chromosome 2 in cell lines (Figure S2B in Additional file 5). The shortest region of amplification, including the complete *HER2 *gene, was delineated to 59,004 bp (chr17:35087594-35146597, hg18 coordinates) comprising *HER2, C17orf37 *, and parts of the *PERLD1 *gene (Figure S2C-E in Additional file 5). Notably, the shortest region of amplification overlap did not include *GRB7*, in contrast to two previous reports [5,20].

Corroborating our previous findings using BAC aCGH [5], we found: 1) four GISTIC regions stratifying HER2-amplified cases based on estrogen receptor (ER)-status of which two were in the same genomic regions identified by BAC aCGH (Figure S2F in Additional file 5), 2) no difference in CN-FGA between ER-negative and ER-positive tumors, 3) 14 recurrent high-level amplifications, excluding chromosome 17, of which eight overlapped directly or were in close proximity to BAC aCGH findings with similar frequencies (Additional file 6), and 4) similar frequency (19%) of amplifications in the centromeric region of chromosome 17, (17p11.1-17q11.1, CEP17-amplification). Additionally, GISTIC was used to delineate the complex pattern of amplification on chromosome 17q, identifying nine regions of gain that all comprised cases with high-level amplification (Additional file 6). Of these nine regions, three overlapped directly with our previous BAC study, and two were in close proximity (< 500 kbp) (Additional file 6). Integration of gene expression data with CN estimates for genes in recurrent amplifications identified numerous genes with significant correlation between mRNA levels and CN estimates (Additional file 6). Notably, the majority of correlated genes were also found to be correlated in our previous study (Additional file 6). In combination with analysis of differential mRNA expression between amplified and non-amplified cases for a set of evaluable genes in the recurrent amplifications (Additional file 6), these analyses confirm the gene dosage dependence of specific genes recurrently amplified in HER2-amplified BC.

To investigate whether observed frequencies of CN gain and loss for the 218 cases were biased due to differences in tumor ploidy and aberrant cell content we calculated the frequency of CN gain and loss in relation to an *in silico *tumor ploidy (GAP-ploidy) for 99 cases analyzed by SNP arrays (Figure 1B). Reassuringly, we found a highly similar pattern of CNAs for these 99 cases (Figure 1B vs. Figure 1A). This finding is in contrast to a recent report using a similar bioinformatical approach in a smaller data set [10].

### Pattern of allelic imbalance in HER2-amplified breast cancer

**Figure 2:**
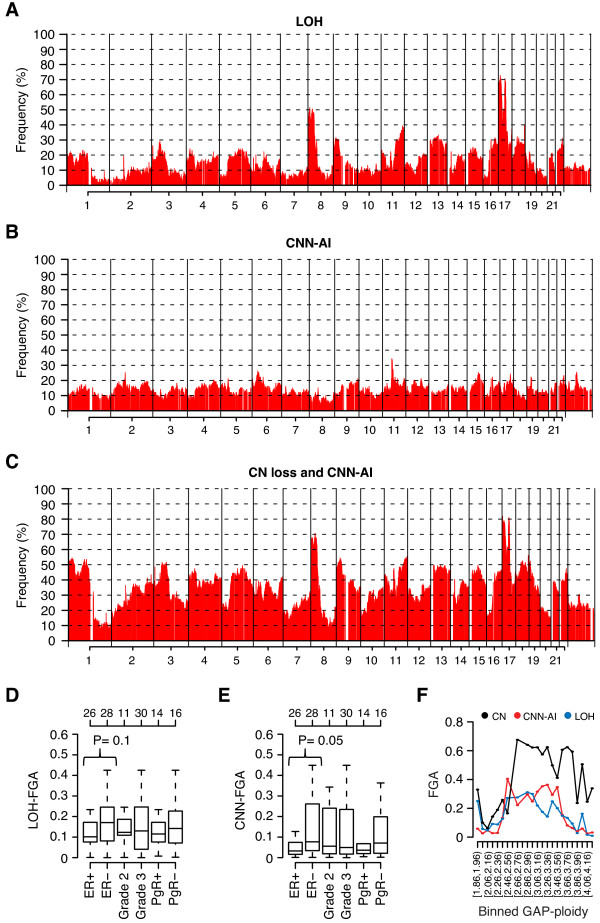
**Frequency of LOH and CNN-AI in HER2-amplified breast cancer**. (**A**) Frequency of LOH across the genome for 99 HER2-amplified cases analyzed by SNP arrays. (**B**) Frequency of CNN-AI across the genome for the 99 cases. (**C**) Frequency of combined CN loss and CNN-AI across the genome for the 99 cases, where CN loss calls were obtained from GAP analysis. (**D**) Fraction of the genome affected by LOH for HER2-amplified cases stratified by different clinicopathological variables. Top-axis indicates number of samples in each group. (**E**) Fraction of the genome affected by CNN-AI for HER2-amplified cases stratified by different clinicopathological variables. Top-axis indicates number of samples in each group. (**F**) Variation of FGA values versus GAP-ploidy (*n *= 99) for CN (black), CNN-AI (red) and LOH (blue). GAP-ploidy estimates were binned in bins of size 0.1 represented by tick marks on the x-axis, and labels for every second bin. For each bin the mean FGA value of included samples are plotted (points) for CN, LOH and CNN-AI. Bins contain different number of samples. *P*-values were calculated using Wilcoxon's test for indicated groups.

The pattern of LOH and CNN-AI in HER2-amplified BC were delineated through GAP analysis of 99 cases analyzed by SNP arrays similarly as described [10]. As expected, LOH was most frequent in regions commonly affected by CN loss, for example, 8p, 17p, 9p, 18q and 13q (Figures 2A and 1B). Interestingly, the frequency of CNN-AI was more evenly distributed across chromosomes and seldom exceeded 20%. The highest CNN-AI frequency in HER2-amplified tumors was observed in a region on 11q close to the centromere (approximately 30% CNN-AI) (Figure 2B). The combination of CNN-AI and CN loss calls derived from GAP analysis (Figure 1B) for the 99 tumors revealed that genomic alterations involving loss of at least one allele were highly frequent (> 60% of cases) in genomic regions on 17p, 8p, and 17q21.3-q22 (Figure 2C). Stratification of the 99 cases into six clinicopathological subgroups showed that the fraction of the genome affected by LOH and CNN-AI were less frequent in HER2-amplified ER-positive tumors compared to ER-negative tumors (*P *= 0.1 and 0.05 respectively, Wilcoxon's test) (Figure 2D, E).

DNA aneuploid HER2-amplified BC has been associated with higher CN-FGA fractions and worse outcome [5]. However, whether such tumors also harbor more LOH or CNN-AI events compared to DNA diploid cases or tumors with low CN-FGA have not been fully elucidated. Among the 99 HER2-amplified tumors we found that cases with lower GAP-ploidy (estimated to be diploid or near diploid), with the exception of hypodiploid cases, harbored less LOH and CNN-AI events, as well as less CNAs compared to cases with higher GAP-ploidy (Figure 2F).

### Pattern of allelic imbalance in HER2-amplified compared to HER2-negative breast cancer

**Figure 3:**
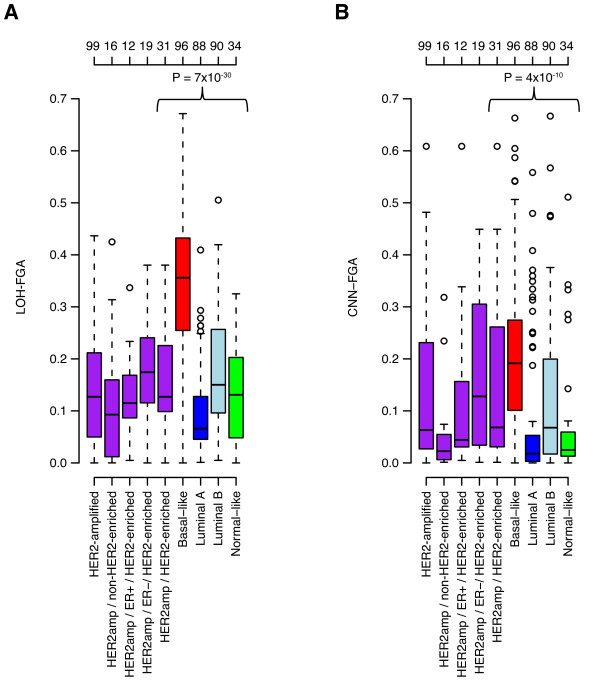
**Fraction of LOH and CNN-AI in HER2-amplified and HER2-negative breast cancer**. (**A**) Fraction of the genome affected by LOH for HER2-amplified cases stratified by PAM50 subtype and ER-status compared to HER2-negative cases stratified by PAM50 subtypes. Top-axis indicates number of samples in each group. A significant difference in LOH-FGA is observed between HER2-amplified and HER2-negative cases stratified by PAM50 subtype (*P *= 7 × 10^-30^, ANOVA). A similar difference was observed when comparing LOH-FGA estimates for all HER2-amplified tumors against HER2-negative cases stratified by PAM50 subtype (*P *= 3 × 10^-33^, ANOVA). (**B**) Fraction of the genome affected by CNN-AI for HER2-amplified cases stratified by PAM50 subtype and ER-status compared to HER2-negative cases stratified by PAM50 subtypes. Top-axis indicates number of samples in each group. A significant difference in CNN-FGA is observed between HER2-amplified and HER2-negative cases stratified by PAM50 subtype (*P *= 4 × 10^-10^, ANOVA). A similar difference was observed when comparing CNN-FGA estimates for all HER2-amplified tumors against HER2-negative cases stratified by PAM50 subtype (*P *= 2 × 10^-10^, ANOVA).

The heterogeneous nature of BC stresses the importance of proper tumor stratification for evaluation of differences between subgroups of BC. To compare the pattern of LOH and CNN-AI in HER2-amplified tumors to other BC subgroups, we performed a similar GAP analysis for the HER2-negative SNP microarray reference data set stratified by PAM50 subtypes (Additional files 1 and 7). Joint analysis of HER2-amplified and HER2-negative cases revealed significant differences between subgroups (Figure 3). Basal-like tumors displayed the highest FGA values for LOH and CNN-AI, followed by luminal B and HER2-amplified/HER2-enriched cases, while luminal A cases displayed the lowest values (Figure 3). Notably, there were no significant differences in LOH-FGA and CNN-FGA values between HER2-enriched tumors stratified by ER-status (*P *= 0.22 and 0.39 respectively, Wilcoxon's test, Figure 3). With the exception for hypodiploid cases (GAP-ploidy clearly < 2), HER2-negative tumors showed increasing fractions of LOH, CNN-AI and CNAs with increasing GAP-ploidy similar to HER2-amplified cases (Additional file 8).

### DNA ploidy and fraction of aberrant tumor cells in HER2-amplified compared to HER2-negative breast cancer

**Figure 4:**
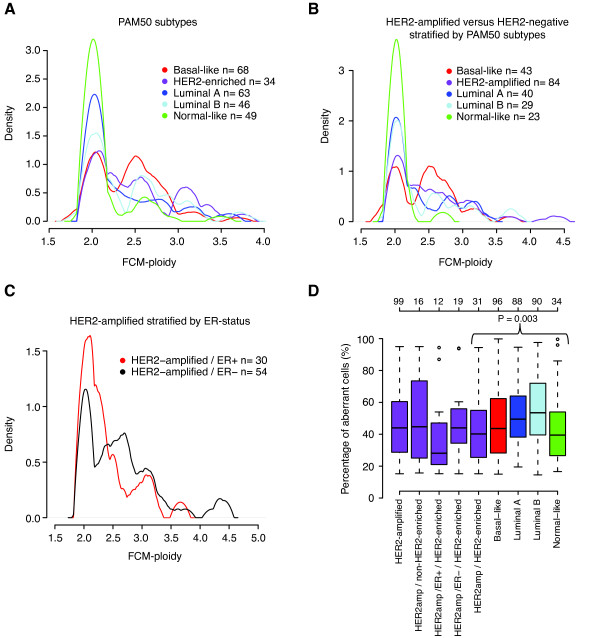
**Tumor ploidy and percentage of aberrant tumor cells in HER2-amplified compared to HER2-negative breast cancer**. (**A**) Distribution of FCM-ploidy across PAM50 subtypes for 260 cases with concurrent gene expression data. Of the 34 HER2-enriched cases, 21 had concurrent BAC aCGH data. 19 of these 21 cases showed amplification of *HER2 *by BAC aCGH, (**B**) Distribution of FCM-ploidy across 219 breast cancer samples grouped as HER2-amplified by BAC aCGH, or HER2-negative by BAC aCGH and further stratified by PAM50 subtype. (**C**) Distribution of FCM-ploidy for 84 HER2-amplified cases stratified by ER-status. (**D**) Distribution of percentage of aberrant cells estimated by GAP analysis of 407 breast cancers across HER2-amplified tumors and HER2-negative tumors stratified by PAM50 subtypes and ER-status. A significant difference in aberrant cell estimates is observed between HER2-amplified and HER2-negative cases stratified by PAM50 subtype (*P *= 0.003, ANOVA). A similar difference was observed when comparing aberrant cell estimates for all HER2-amplified tumors against HER2-negative cases stratified by PAM50 subtype (*P *= 0.02, ANOVA). In A to C, curves were generated by an Epanechnikov smoothing kernel with 0.08 smoothing bandwidth.

To investigate the pattern of DNA ploidy in HER2-amplified tumors compared to other subgroups of BC we first analyzed FCM derived DNA histograms from 338 external cases with connected gene expression and BAC aCGH data (Table S1 in Additional file 1). Stratification of the 338 tumors by PAM50 subtypes revealed differences in the distribution of FCM-ploidy between subtypes. For instance, the luminal A and normal-like subtypes showed the highest proportions of FCM-ploidy values close to 2N (most often diploid) (Figure 4A). In contrast, basal-like tumors showed a clear bimodal appearance with peaks at 2N and around 2.5N (Figure 4A). Furthermore, the HER2-enriched subtype showed a high proportion of cases with FCM-ploidy around 3N, while luminal B tumors showed pronounced peaks at 2N and around 2.6N (Figure 4A). Similar patterns were observed when comparing FCM-ploidy from 84 HER2-amplified tumors with 150 cases without amplification of *HER2 *(determined by BAC aCGH) stratified by PAM50 subtypes (Figure 4B). An exception was that the peak at 3N was less pronounced in HER2-amplified cases when viewed as a single entity (Figure 4B) compared to HER2-enriched cases (Figure 4A). GAP-ploidy estimates for 407 cases (99 HER2-amplified and 308 HER2-negative with subtype classification) analyzed by SNP arrays showed patterns similar to DNA-FCM results. Again, a bimodal distribution was observed for basal-like tumors, a predominantly diploid pattern for luminal A and normal-like tumors, and a more complex pattern for luminal B and HER2-amplified tumors with peaks at 3N and higher (Figure S5A in Additional file 9).

Interestingly, stratification of the 84 FCM analyzed HER2-amplified cases by ER-status revealed differences, in that ER-negative cases displayed a more pronounced bimodal distribution of FCM-ploidy estimates similar to HER2-negative basal-like tumors, while the 3N peak was most pronounced in HER2-amplified ER-positive cases (Figure 4C). A similar bimodal distribution of GAP-ploidy estimates was observed for 28 annotated ER-negative HER2-amplified tumors analyzed by SNP arrays, although with a shift towards 3N for the second peak (Figure S5B in Additional file 9). In contrast, the concordance between GAP-ploidy and FCM-ploidy distributions from the two different patient cohorts were poorer for HER2-amplified ER-positive tumors (Figure 4C and Figure S5B in Additional file 9).

Although quantitative DNA FCM analysis can accurately detect and quantify DNA aneuploid cell populations, it cannot delineate the proportion of, for example, diploid cancer cells versus diploid non-tumor cells. To investigate the fraction of aberrant cells in HER2-amplified and HER2-negative BC stratified by PAM50 subtypes, we compared aberrant cell estimates obtained from GAP analysis for 99 versus 308 cases analyzed by SNP microarrays, respectively. Small differences in aberrant cell estimates between subtypes were observed (*P *= 0.003, ANOVA), with the luminal subtypes, generally associated with ER-positivity, showing the highest estimates (*P *= 0.00004, Wilcoxon's test, luminal subtypes versus remaining subgroups combined) (Figure 4D). In contrast, no difference in aberrant cell percentages was observed for stratification of all HER2-amplified cases, or HER2-enriched tumors by ER-status (*P *= 0.95 and 0.14 respectively, Wilcoxon's test).

### Tumor subclonality in breast cancer revealed by SNP array analysis

**Figure 5:**
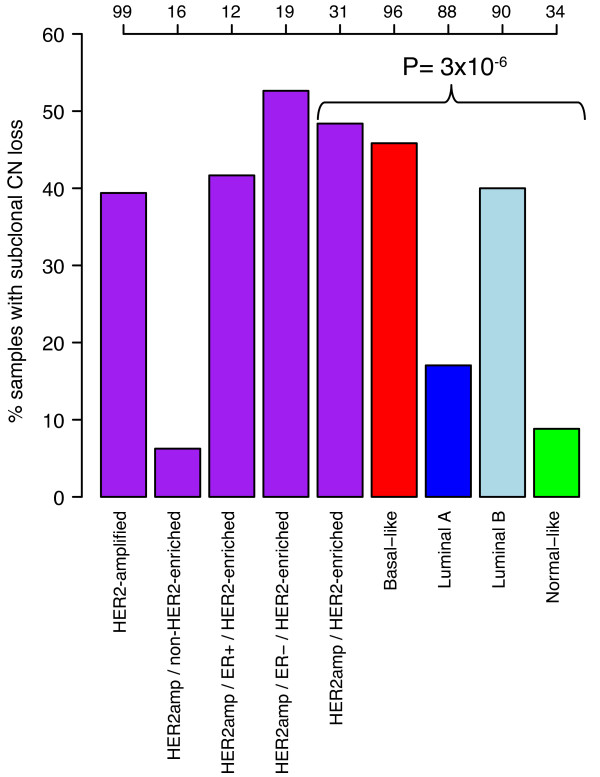
**Prevalence of subclonal CN loss in breast cancer**. Prevalence of subclonal CN loss was estimated in 99 HER2-amplified cases and 308 HER2-negative cases with PAM50 subtype classification analyzed by SNP arrays revealing differences in the frequency of subclonal CN loss events between subgroups. Top-axis indicates total number of cases for each bar. *P*-value was calculated using the Chi-square test for indicated groups.

BC often display extensive intra-tumor heterogeneity regarding, for example, cell size, morphology, proliferation and genomic alterations [39-41]. To a certain extent, aCGH and SNP arrays allow detection of CNAs that differ in prevalence among analyzed cells. To investigate the prevalence of subclonal CN events in HER2-amplified versus HER2-negative BC, we employed a conservative approach limited to larger CN deletions only (see Material and methods). In the 99 HER2-amplified BCs analyzed by SNP arrays, this approach identified 39 cases (39%) with subclonal CN loss. By comparison, in the 308 HER2-negative cases 98 samples (32%) were identified as having subclonal CN loss. Differences in the prevalence of subclonal CN loss events were observed between HER2-amplified/HER2-enriched and HER2-negative cases stratified by molecular subtype (*P *= 3 × 10^-6^, Chi-square test). HER2-amplified/HER2-enriched cases together with basal-like tumors showed the highest prevalence of subclonal CN loss, followed by luminal B tumors, while luminal A and normal-like tumors displayed the lowest fractions (Figure 5). Subclonal CN loss was associated with higher fractions of LOH, CNN-AI, CNAs and higher GAP-ploidy for HER2-amplified, HER2-amplified/HER2-enriched, luminal A and luminal B cases (*P *< 0.05, Wilcoxon's tests). For basal-like tumors subclonal CN loss was significantly associated with higher CN-FGA fractions and higher GAP-ploidy (*P *< 0.0001, Wilcoxon's test), while only weakly associated with higher LOH-FGA fractions (*P *= 0.11, Wilcoxon's test).

### High-level amplification events appear as monoallelic in HER2-amplified breast cancer

**Figure 6:**
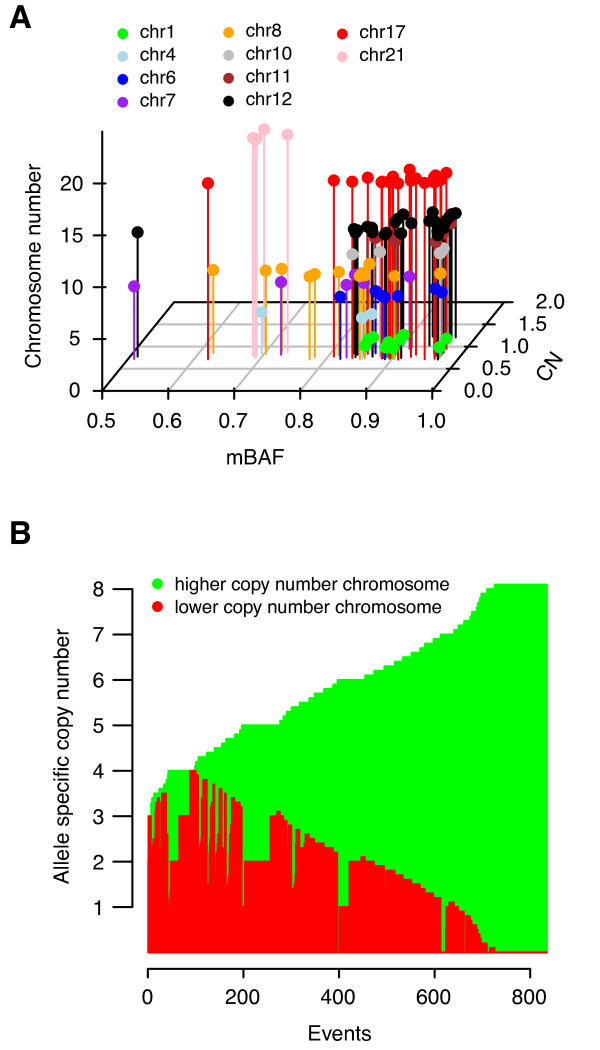
**Monoallelic amplification in HER2-amplified breast cancer**. (**A**) High-level amplifications predominantly show amplification of one parental chromosome in HER2-amplified breast cancer. Partitioned mBAF [12] estimates are plotted against corresponding partitioned CN values for 108 genomic segments > 50 kbp in size and with a partitioned log2ratio > 0.7 from cases in the Lund-HER2-SNP set, representing high-level amplification segments in this data set. The color and height of segments on the z-axis indicate chromosome location. BAF estimates range between 0 and 1 reflecting the proportion of the two parental chromosomes for a given SNP. Due to the symmetry of BAF profiles for current genome-wide SNP arrays, BAF profiles may be reflected along the central 0.5 axis into mBAF estimates [12]. In a normal sample mBAF values close to 0.5 represent a heterozygous state (AB) for a given SNP, whereas values close to 1 represent a homozygous state (AA or BB). In tumor samples allelic imbalances are seen as a deviation of heterozygous SNPs from 0.5. The amplitude of the deviation is a function of the type of aberration, fraction of aberrant cells carrying the aberration and tumor ploidy. Thus, for amplification events in tumor samples higher mBAF values indicate additional copies of one of the two parental chromosomes in the amplified region. (**B**) Distribution of estimated allele specific copy numbers from GAP analysis for 835 events in 90 of 99 samples analyzed by SNP arrays, > 2 Mbp in size and with a total CN > 3 compared to the GAP-ploidy for respective sample. The latter means that if the GAP-ploidy of a sample is 2.58 an event must have a total CN > 5.58 to be included. For each event on the x-axis allele specific copy numbers for the two parental chromosomes are shown as green and red bars. The total CN is the sum of the height of the red and green bars, and is truncated at eight by GAP. GAP returns allele specific copy numbers as numerical values, for example, 2.5, for possible identification of sub-clonal events, thus the steps in the plot are not discrete.

Genes residing in regions subjected to genomic amplification are oncogene candidates and gene amplification may occur through different mechanisms. SNP arrays offer the possibility to dissect the gene amplification process on an allele specific level as demonstrated by LaFramboise *et al*. [42]. In a panel of lung cancer tumors and cell lines essentially all amplification events appeared to be monoallelic; that is, gene amplification exclusively involved only one of the two parental chromosomes [42]. To investigate whether this observation stands true also in HER2-amplified BC, we aimed to identify more focal high-level amplification events in the Illumina Lund-HER2-SNP set by selecting only smaller segments (> 50 kbp) with a high partitioned CN log2ratio (> 0.7). These criteria identified 108 segments among the 26 cases. Integration of partitioned CN and mBAF values [12] for these 108 segments showed that the absolute majority of them displayed simultaneously high CN and mBAF values (Figure 6A). These results suggest that high-level amplifications involve only one of the parental chromosomes also in HER2-amplified BC.

In contrast to the less frequent and more focal high-level amplifications, amplifications involving a larger part of a chromosome arm but with a lower number of additional gene copies are more frequent in BC. To investigate whether such amplification events also preferentially target one parental chromosome, we identified 90 cases from the 99 HER2-amplified tumors analyzed by SNP arrays that harbored 835 genomic regions > 2 Mbp and with a total CN > 3 compared to the estimated GAP-ploidy for respective sample. Similar to high-level amplifications, the majority of identified events displayed amplification of predominantly one of the two parental chromosomes (Figure 6B).

## Discussion

HER2+ BC represents an important clinical subgroup of the disease due to availability of effective targeted therapy both in the adjuvant and metastatic setting. Clinically, the subgroup is defined by *HER2 *gene amplification and/or protein overexpression; however, genome-wide molecular analyses have shown that BC with genomic amplification of HER2 (HER2-amplified BC) is heterogeneous with regards to gene expression patterns, CNAs and outcome [4,5,20]. Thus, further characterization of HER2-amplified tumors at the gene level may have implications for improved diagnosis, prognosis and prediction.

Here we report the first integrated analysis of CNAs and AI in a large cohort of HER2-amplified BC profiled by high-density genomic microarrays, allowing a comprehensive description of the genomic landscape of CNAs, amplifications, LOH and CNN-AI. When comparing results to our previous study of 200 HER2-amplified tumors profiled by BAC aCGH [5], we corroborated several previous findings regarding, for example, amplifications, and found a striking similarity in the overall pattern of CN gain and loss. By comparing significant CNAs identified by GISTIC analysis in the current and former study [5], we were able to define a core set of genomic regions commonly affected by CN gain and loss in HER2-amplified BC across different genomic microarray platforms that may serve as a list of potential targets for further studies (Additional file 4). Differences between the two studies may be explained by usage of different array platforms, data analysis methods and cohort composition. Importantly, however, the concordance between our two studies emphasizes that evaluation of CNAs in a heterogeneous subgroup such as HER2-amplified BC needs to be performed in large sample sets in order to pinpoint recurrent alterations.

Genome-wide analyses of LOH, CNN-AI, tumor ploidy, fraction of aberrant cells and subclonal CN events utilizing genomic microarrays have been scarce in BC due to the often high sample complexity, lack of appropriate analysis methods and low sample numbers. In the current study, we applied GAP [13] to SNP array data in combination with conventional DNA-FCM to analyze genomic alterations on an allele specific level, patterns of tumor ploidy, tumor subclonality and fraction of aberrant cells in a large set HER2-amplified and HER2-negative tumors stratified by molecular subtype. In HER2-amplified cases as well as HER2-negative subgroups the pattern of LOH was, as could be expected from the LOH definition, strongly associated with the pattern of CN loss (Figures 1 and 2, Additional file 7). In contrast, CNN-AI events were more evenly distributed across chromosomes in HER2-amplified tumors, seldom exceeding > 20% in frequency and not targeting specific genomic regions (Figure 2). Interestingly, a similar low and evenly distributed CNN-AI pattern was also observed in HER2-negative luminal A, luminal B and normal-like tumors (Additional file 7). In contrast, basal-like tumors showed slightly higher frequencies potentially explained by a higher frequency of triploid cases (3N). This suggests that CNN-AI appears as a less frequent genome-wide additive event in the majority of breast cancers. Moreover, in relation to other BC subtypes the patterns of LOH and CNN-AI were similar to findings by Van Loo *et al*. [10], and also mimicked the general pattern of CN-FGA reported for BC gene expression subtypes [5,36]. However, based on our joint analysis of 407 HER2-amplified and HER2-negative cases, we were not able to corroborate previously reported subtype specific pattern of aberrant cell estimates [10] (Figure 4D). This discrepancy between studies warrants further investigation, but indicates that these types of estimations may be difficult to systematically reproduce. Interestingly, the finding in the current study that HER2-amplified and predominantly ER-negative basal-like tumors show lower aberrant cell estimates is consistent with observations of considerable lymfocytic infiltration in these subtypes [43-45]. In agreement with observations in lung cancer [42] we found that amplifications in HER2-amplified BC were essentially monoallelic, as amplification preferentially targeted one of the two parental chromosomes (Figure 6). This form of amplification may be a mechanism for targeting activating oncogene mutations and has previously been observed on an individual gene level [46,47]. The full significance of this putative mechanism, however, remains to be investigated in more detail using, for example, rapidly evolving sequencing techniques.

Aneuploidization is one of the most common properties of cancer and has generally been associated with worse prognosis and more advanced disease [48]. In support of an overall higher genomic complexity for aneuploid BC, we found that increasing GAP-ploidy was associated with higher fractions of LOH, CNN-AI and CNAs, as well as higher occurrence of subclonal CN loss events irrespective of BC subtype (Figure 2F and Additional file 8). Not surprisingly, the patterns of DNA ploidy, subclonal CN events, fractions of LOH, CNAs and CNN-AI across HER2-amplified and HER2-negative tumors appear consistent with the overall prognosis for the subgroups. For instance, luminal A and normal-like tumors, which generally display the best outcome, are more frequently diploid and less complex. In contrast, basal-like, HER2-amplified and luminal B cases display more complex patterns in line with their poorer outcome and often higher stage [24,36]. Although both GAP and a similar method termed Allele-Specific Copy number Analysis of Tumors [10] allow estimation of *in silico *tumor ploidy from SNP array data, both methods have difficulties in analyzing certain types of samples [10,13]. To get a more unbiased analysis of the pattern of DNA ploidy across BC subtypes, we used quantitative DNA FCM data for 338 unrelated BCs also analyzed by gene expression microarrays and BAC aCGH. Using this large sample set we were able to corroborate several findings by Van Loo *et al*. [10], as well as results from our GAP analysis, for example, showing that the molecular BC subtypes display different patterns of tumor DNA ploidy. Shifts between FCM and GAP-ploidy peak positions, exemplified by HER2-negative basal-like tumors (Figures 4A and S5A in Additional file 9), may be explained by that the latter estimation aims to account for normal cell contamination, while the former represents a mere total DNA summarization.

Interestingly, the bimodal distribution of tumor ploidy displayed by HER2-negative basal-like tumors was also observed in ER-negative HER2-amplified tumors, and in ER-negative tumors in general irrespective of subtype (data not shown). These findings imply that the evolutionary hypothesis for basal-like tumors suggested by Van Loo *et al*. [10], of a reduction from a diploid to a partial haploid state followed by whole-genome duplication, is not limited to a specific molecular subtype but appears to be more general for ER-negative BC. This apparently more general difference in DNA ploidy patterns between ER-positive and negative BC most likely explain differences in LOH and CNN-AI fractions observed between subgroups/subtypes of HER2-amplified BC as, for example, ER-negative tumors are overrepresented in the HER2-enriched subtype. The HER2-enriched subtype has been found to often comprise the majority of HER2-amplified cases in gene expression studies. However, based on findings from several recent studies, including the current one, it appears clear that 1) the HER2-enriched subtype identified by different single sample predictors is not synonymous with the clinically defined HER2+ subgroup, 2) the subtype includes a notable fraction of HER2-amplified ER-positive cases, 3) HER2-amplified cases are found in all gene expression subtypes at varying frequencies, and 4) HER2-negative cases are found in the HER2-enriched subtype [5,20,24,25]. As an example of the latter, we found that 7.5% of samples in the 346-sample HER2-negative SNP reference set were classified as HER2-enriched by the PAM50 single sample predictor.

In summary, the comprehensive analysis presented herein confirms and extends several findings about the reported molecular subtypes of BC, but also emphasizes the strong association of different types of genomic aberrations with tumor DNA aneuploidy, irrespective of subtype. The molecular BC subtypes have repeatedly been shown to display different CNAs [36,49,50], and, lately, also differences in fractions of LOH and CNN-AI [10]. We demonstrate that tumors harboring few CNAs typically also display less LOH, less CNN-AI, lower tumor ploidy and less frequent occurrence of subclonal events, pointing towards an overall lower complexity irrespective of subtype.

## Conclusions

We have conducted a comprehensive survey of genomic alterations in the largest cohort of HER2-amplified breast cancer cases profiled by aCGH and SNP arrays reported to date. Taken together, the results presented here provide a comprehensive view of the landscape of genomic alterations including CNAs, LOH, CNN-AI and tumor DNA ploidy occurring in HER2-amplified tumors in context of other BC subgroups. Furthermore, our results emphasize that analysis of genomic alterations in heterogeneous subgroups, such as HER2-amplified BC, need large sample numbers in order to draw more definite conclusions.

## Abbreviations

aCGH: array-based Comparative Genomic Hybridization; AI: allelic imbalance; BAF: B allele frequency; BC: breast cancer; CN: copy number; CNN-AI: copy number neutral allelic imbalance; CNA: copy number alteration; ER: estrogen receptor; FCM: flow cytometry; FGA: fraction of the genome altered; GAP: genome alteration print; GISTIC: genomic identification of significant targets in cancer; HER2: Human epidermal growth factor receptor 2; LOH: loss of heterozygosity; SNP: single nucleotide polymorphism

## Competing interests

JS and ÅB have received honoraria from Roche. The other authors declare that they have no competing interests.

## Authors' contributions

JS conceived of the study and performed microarray data analysis with support by GJ and MR. BB performed FCM analysis. JS wrote the manuscript with the assistance of GJ, MR, BB and ÅB. All authors read and approved the final manuscript.

## Supplementary Material

Additional file 1 **Identification of HER2-amplified cases and patient and tumor characteristics for reference data sets**. A pdf file containing figure S1 showing *HER2 *CN estimates for identified HER2-amplified cases from public repositories, and a table (S1) of patient and tumor characteristics for HER2-amplified, HER2-negative tumors in the SNP reference breast cancer data set, and the 338 tumors analyzed by FCM. Click here for file

Additional file 2 **Supplementary Methods**. A Word document containing supplementary information about used methods and data processing. Click here for file

Additional file 3 **Significant GISTIC regions identified from analysis of 218 *HER2*-amplified breast cancers**. An Excel table presenting significant GISTIC regions and frequency of amplification in these regions, as well as sample identifiers for the 218 primary tumors used for GISTIC analysis. Click here for file

Additional file 4 **Recurrent GISTIC regions in *HER2*-amplified breast cancer**. An Excel table presenting GISTIC regions that overlaps with a previous study of 200 HER2-amplified cases analyzed by BAC aCGH. Click here for file

Additional file 5 **CNAs in HER2-amplified breast tumors and cell lines**. A pdf file containing six figures, S2A-F, showing the overlap of GISTIC regions identified in the current and a former BAC aCGH study, pattern of CNAs in 16 HER2-amplified breast cancer cell lines, shortest region of amplification including the *HER2 *gene, HER2-amplified cases defining the centromeric and telomeric breakpoints for the shortest region of amplification analysis, and GISTIC regions stratifying HER2-amplified breast cancer based on ER-status, respectively. Click here for file

Additional file 6 **Recurrent amplifications in HER2-amplified breast cancer**. An Excel table describing recurrent amplifications in the 218 HER2-amplified cases, and the integration with concomitant gene expression data for genes in amplified regions. Additionally, the overlap between recurrent amplifications with a previous study of 200 HER2-amplified cases analyzed by BAC aCGH is presented. Click here for file

Additional file 7 **Frequency of LOH and CNN-AI in HER2-negative breast cancers analyzed by GAP and stratified by PAM50 subtypes**. A pdf file containing four figures, S3A-D showing frequency of LOH (upper panel) and CNN-AI (lower panel) for HER2-negative tumors classified as basal-like, luminal A, luminal B, and normal-like using PAM50. Click here for file

Additional file 8 **Variation of FGA values versus GAP-ploidy for HER2-amplified and HER2-negative breast cancers**. A pdf file containing five figures, S4A-E, showing the pattern of LOH-FGA, CNN-FGA and CN-FGA for HER2-amplified cases, HER2-negative basal-like tumors, HER2-negative luminal A tumors, HER2-negative luminal B tumors and HER2-negative normal-like tumors respectively. Click here for file

Additional file 9 **Tumor ploidy for HER2-amplified cases estimated by GAP analysis**. A pdf file containing two figures, S5A-B, showing the distribution of GAP-ploidy estimates for 407 HER2-amplified and HER2-negative cases stratified according to subtype, and HER2-amplified cases stratified by ER-status respectively. Click here for file
