# Supplementary material for: Landscape of somatic allelic imbalances and copy number alterations in HER2-amplified breast cancer
Source: Breast Cancer Res. 2011 Dec 14;13(6):R129. doi: 10.1186/bcr3075 (PMC3326571; doi:10.1186/bcr3075)
Supplement: Additional file 1 — Identification of HER2-amplified cases and patient and tumor characteristics for reference data sets. A pdf file containing figure S1 showing HER2 CN estimates for identified HER2-amplified cases from public repositories, and a table (S1) of patient and tumor characteristics for HER2-amplified, HER2-negative tumors in the SNP reference breast cancer data set, and the 338 tumors analyzed by FCM. [file bcr3075-S1.PDF]

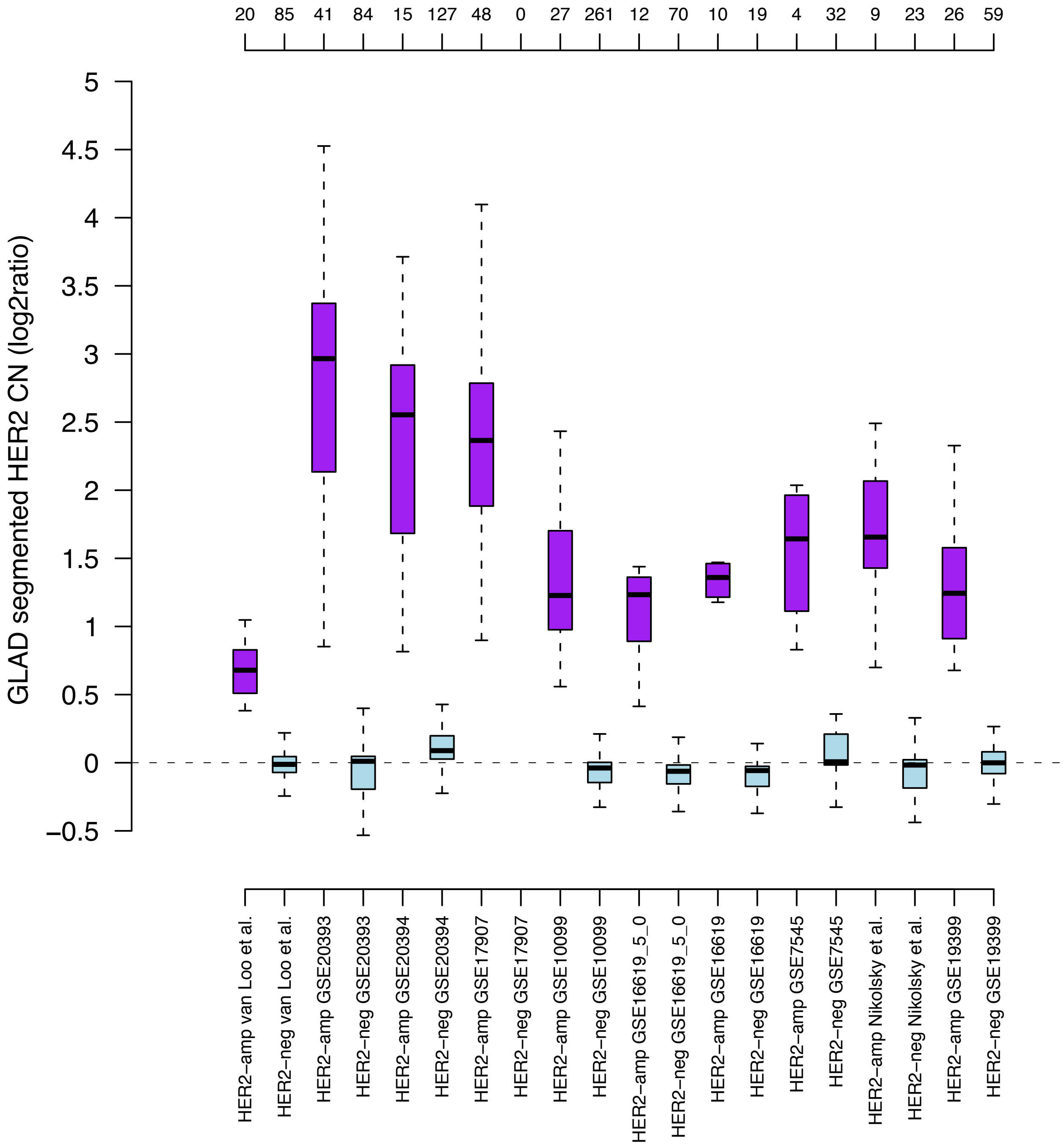

Supplementary Figure 1. Identification of HER2-amplified cases from public repositories. Boxplots of GLAD segmented CN estimates for *HER2* from cases identified as having a 17q12 amplification including *HER2* (HER2-amplified, purple), compared to remaining cases from respective data set (light blue). Top-axis indicate number of samples in each boxplot, representing the number of processed and quality filtered samples from each data set. Notably, *HER2* CN levels vary significantly between data sets due to usage of different aCGH / SNP platforms: Illumina (van Loo et al.), Agilent (GSE20393, GSE20394, GSE17907) and Affymetrix (GSE10099, GSE16619, GSE7545, Nikolsky et al., and GSE19399). Consequently, a single log2 ratio value is not optimal for identification of amplified samples.

Table S1. Characteristics of HER2-amplified tumors with concurrent gene expression data, the HER2-negative SNP reference set and the 338 breast tumors analyzed by FCM.

	HER2-amplified with gene expression*	HER2-amplified with GEX profiled by SNP*	HER2-negative SNP reference set	FCM cohort (n=338)	
				Cases with GEX**	Cases with aCGH**
Total number	99	47	346	282	234
Number HER2-amplified SNP/aCGH	99	47[A]	0	28	84
Number HER2-negative SNP/aCGH	0	0	346	150	150
PAM50 subtypes					
Basal-like	6	4	96	68	44[B](43)
HER2-enriched	72	31	26	34[C]	21[B] (2)
Luminal A	5	2	88	63	41[B] (40)
Luminal B	9	5	90	46	31[B] (29)
Normal-like	6	4	34	49	28[B] (23)
Unclassified	1	1	12	22	13[B] (13)
ER-status					
ER-negative	50	25	NA	68	101
ER-positive	45	22	NA	109	128

* Cohort representing a subset of the 218 primary tumors obtained from public repositories.

** The two sub-cohorts partly overlap, meaning that a large portion of samples have both gene expression and BAC aCGH data available.

[A] All cases profiled by SNP arrays only.

[B] The number within parentheses indicates the number of HER2-negative tumors assigned to respective PAM50 subgroup.

[C] Of 34 HER2-enriched cases, 21 had concurrent BAC aCGH data. 19 of these 21 cases displayed amplification of HER2 by BAC aCGH.
