# Supplementary material for: Landscape of somatic allelic imbalances and copy number alterations in HER2-amplified breast cancer
Source: Breast Cancer Res. 2011 Dec 14;13(6):R129. doi: 10.1186/bcr3075 (PMC3326571; doi:10.1186/bcr3075)
Supplement: Additional file 2 — Supplementary Methods. A Word document containing supplementary information about used methods and data processing. [file bcr3075-S2.DOCX]

**Supplementary Methods**

**Estimation of copy number and allele estimates for Affymetrix SNP data**

Copy number (CN) and B Allele Frequency (BAF) estimates were generated specifically for the different Affymetrix data sets using CRMAv2 [29] and ACNE [30] in combination as outlined in the ACNE vignette (http://www.aroma-project.org/). All data sets were mapped to the hg18 genome build. Briefly, raw data from Affymetrix CEL files were: 1) calibrated for allelic cross talk using the AllelicCrosstalkCalibration function with the CRMAv2 model parameter, 2) normalized for nucleotide-position probe sequence effects using the BasePositionNormalization function with target parameter set to zero, and 3) probe summarized using the ACNE NmfSnpPlm function as outlined specifically for each data set below.

Specifically, for each data set:

- GSE7545 (Affymetrix 250K Nsp and Sty): CEL files were obtained from Gene Expression Omnibus (GEO) [28]. ACNE [30] analysis was performed according to vignette (http://www.aroma-project.org/). 276 HapMap cases obtained from GSE5173 (GEO) were used as specific reference set in the NmfSnpPlm function to generate copy number and B Allele Frequency estimates. Only Nsp chip data were used in further analyses.
- GSK Breast (Affymetrix 250K Nsp and Sty): CEL files were obtained as described on the TumorScape website, http://www.broadinstitute.org/tumorscape/pages/portalHome.jsf. ACNE analysis was performed according to vignette. 276 HapMap cases obtained from GSE5173 were used as specific reference set in the NmfSnpPlm function to generate copy number and B Allele Frequency estimates. Only Nsp chip data were used in further analyses.
- GSE16619 (Affymetrix 250K Nsp and Sty): CEL files were obtained from GEO. ACNE analysis was performed according to vignette. 276 HapMap cases obtained from GSE5173 were used as specific reference set in the NmfSnpPlm function to generate copy number and B Allele Frequency estimates. Only Nsp chip data were used in further analyses.
- Nikolsky et al. [16] (Affymetrix 250K Sty, generated at Broad Institute): CEL files obtained from TumorScape website (http://www.broadinstitute.org/tumorscape/pages/portalHome.jsf). ACNE analysis was performed according to vignette. 382 normal samples obtained from the TumorScape website (http://www.broadinstitute.org/tumorscape/pages/portalHome.jsf) were used as specific reference in the NmfSnpPlm function to generate CNs estimates. BAF estimates were generated by using the NmfSnpPlm function with 276 HapMap samples obtained from GSE5173 as specific reference as this yielded significantly better data quality compared to Broad normal samples.
- GSE19399 (Affymetrix 250K Sty, generated at Broad Institute): CEL files were obtained from GEO. ACNE analysis was performed according to vignette. 382 normal samples obtained from the TumorScape website (http://www.broadinstitute.org/tumorscape/pages/portalHome.jsf) were used as specific reference in the NmfSnpPlm function to generate CN estimates. BAF estimates were generated by using the NmfSnpPlm function with 276 HapMap samples obtained from GSE5173 as specific reference as this yielded better data quality compared to Broad normal samples.
- GSE10099 and GSE13696 (Affymetrix 100K Hind and Xba). CEL files were obtained from GEO. ACNE analysis was performed according to vignette. 270 HapMap cases obtained from the HapMap website (www.hapmap.org) were used as specific reference set in the NmfSnpPlm function to generate CN and BAF estimates.
- GSE16619 (Affymetrix GenomeWideSNP 5.0). CEL files were obtained from GEO. CRMAv2 [29] normalization was performed according to vignette (http://www.aroma-project.org/) to generate CN estimates, using 30 HapMap trios obtained from the Affymetrix website (www.affymetrix.com) as reference.

**Estimation of copy number and allele estimates for Illumina SNP data**

SNP annotations, CN and BAF estimates were updated / generated specifically for the different Illumina data sets as outlined below.

- Van Loo et al. [10] (Illumina 109K): SNP positions were updated to hg18 using the SNP126 database. Matched normal samples were not used.
- GSE11977 (Illumina 550K): CN and BAF estimates were obtained from tQN-normalized data from Staaf et al. [21]. SNP positions were given in hg18 coordinates.
- Lund-HER2-SNP set (Illumina Omni 1M quad): SNP positions were updated to hg18 based on mapping information obtained from the UCSC Genome Browser (http://genome.ucsc.edu/).
- Lund-HER2-SNP set (Illumina Omni 2.5M quad): SNP positions were updated to hg18 based on mapping information obtained from Illumina. BAF estimates were tQN-normalized as described ([21] and http://baseplugins.thep.lu.se/wiki/se.lu.onk.IlluminaSNPNormalization) due to asymmetry in GenomeStudio BAF estimates.

**Preprocessing of Agilent 244K data sets:**

Agilent 244K aCGH data sets were preprocessed prior to GLAD segmentation as outlined below and updated to hg18 positions based on platform annotations from GEO:

- GSE20394. Normalized log2ratio values obtained from GEO.
- GSE17907. Pre-partitioned log2ratio values obtained from GEO. **This data set was not GLAD segmented**.
- GSE20393. Normalized log10ratio values obtained from supplementary raw data files available through GEO and converted to log2 scale.

**GLAD segmentation**

GLAD segmentation was performed using the Bioconductor GLAD package for R (www.bioconductor.org). Default parameters were used with the exception of the bandwith parameter that was set to 1 for improved speed. Prior to segmentation missing values were removed. GLAD segmentation output was reformatted into a format similar to output obtained from the R DNAcopy package (CBS) for downstream analysis.

**CBS segmentation**

The Illumina Omni 2.5M CN data was segmented using CBS [32] implemented in the DNAcopy R package with an α = 0.001. Change points were analyzed using the SDundo option in CBS with a SD threshold of 1.

**Integration of partitioned CN and mBAF**

Individually partitioned CN and mBAF values (reflected BAF estimates, see [12]) for cases analyzed by SNP arrays were integrated using R scripts, with the aim to assign a partitioned mBAF value to an existing CN segment, and in certain instances to create new CN segments based on mBAF data. For each sample and CN segment, assignment of a corresponding mBAF value was made based on the number of partitioned mBAF segments identified within the actual CN segment:

1) A single partitioned mBAF segment existed. If only a single partitioned mBAF value existed this segment was selected to represent the CN segment.

2) Multiple mBAF segments existed. If one segment was predominant (>95% of probes) this segment was selected to represent the CN segment. Otherwise, the original CN segment was broken up into smaller CN segments representing the different mBAF segments within it, assigning new CN values (mean values) based on included probes log2ratios. mBAF segments < 5% in size of the original CN segment were excluded in the creation of new CN segments.

**Merging of partitioned genomic profiles from different data sets**

Merging of partitioned genomic tumor profiles from different data sets was performed similarly as outlined in Gunnarsson et al. [34] with the following changes: 1) basic resolution was set to 10000bp, 2) actual breakpoints from segmentation analysis of samples were added to the 10000bp probe set, and 3) missing values were imputed by assigning the value of the closest probe (in base pair) with a valid segment value to a probe displaying a missing value. The final probe set comprised 394743 probes for chromosomes 1 to 22 and X.

**GISTIC Analysis**

Segmented CN profiles from 218 HER2-amplified tumors merged to the common 10000bp probe set were subjected to GISTIC analysis. GISTIC analysis was performed using the GenePattern server (<http://www.broadinstitute.org/cancer/software/genepattern/>) using version 3 of GISTIC. GISTIC thresholds for gain and loss were set to log2ratio ±0.12. Segments < 9 probes in size were joined by the GISTIC algorithm, and chromosome X was excluded from analysis. A residual q-value cut-off of 0.05 was used to identify significant GISTIC regions. The cnv file required by GISTIC was created by matching probes to the ”variation.hg18.v9.mar.2010.txt” file obtained from the Database of Genomic Variants (http://projects.tcag.ca/variation/).

**GAP analysis**

GAP analysis was performed using integrated CN and AI data merged to the common probe set as described above. R-scripts were used to transform the partitioned and integrated data into a format suitable for the “CopyNumber_and_Genotype” GAP function obtained from authors website [13]. The in silico tumor ploidy was calculated from the output of the above function similarly as described [13]. The 218 primary tumors collected from public repositories were analyzed separately from other cases.

**Gene expression analyses**

Gene expression data were available for samples in GSE20394, GSE10099, Van Loo et al. and GSE17907. Normalized and mean-centered gene expression data for GSE10099 (comprising samples from GSE2034 and GSE5327) were obtained from GOBO [26]. Normalized gene expression data for GSE20394 and Van Loo et al. were obtained from the Stanford Microarray Database (http://smd.stanford.edu). cDNA probe annotations were updated to Unigene Build 221 through ACID (http://bioinfo.thep.lu.se/acid) using probe IMAGE identifiers. For each data set separately, cDNA probes were filtered for >70% present data points (presence), and remaining probes were mean-centered across tumors.

Affymetrix U133 2plus CEL files for GSE17907 were obtained from GEO, normalized using MAS5 and updated for probe annotations as described [26]. GSE17907 MAS5 normalized data was joined with additional breast cancer samples analyzed by Affymetrix U133 2plus arrays obtained from GEO (GSE13787, GSE16391, GSE16446, GSE18728, GSE19697, GSE3744, GSE5460, GSE6532, GSE7904, GSE9195) or CaArray (PMID=17317830, http://caarraydb.nci.nih.gov/caarray/) that were preprocessed similarly. The final set comprised 680 unique breast cancer samples. Affymetrix probe sets in the 680-sample set were subsequently mean-centered across all tumors. Finally, U133A probe sets were extracted from the mean-centered data and used in further analyses.

Data sets were classified into molecular subtypes using the PAM50 [24] molecular subtypes based on Pearson correlation to centroids obtained from Weigelt et al. [25]. Samples with highest correlation < 0.2 to any gene expression centroid were denoted unclassified. If multiple probes were present for a gene, the probe with the highest standard deviation across samples was chosen to represent the gene in correlation analysis with PAM50 centroids.

Correlation of gene expression data with genomic aberrations (recurrent amplifications, core GISTIC regions) for HER2-amplified cases was performed similarly as described [27]. Specifically, matched gene expression and CN matrices for cases with connected gene expression data were created by adding gene expression data for samples on a data set specific basis. Thus, the different gene expression platforms were not merged to a common data set. Consequently, gene expression levels for a gene in a given sample in the gene expression matrix used for correlation is relative to the mean centering for the data set the sample belong to. Matching of gene expression data was made on gene identifier (symbol). If multiple probes existed for a gene the average log2ratio expression was used. The corresponding CN value for a gene in a given sample was taken as the partitioned log2ratio for the amplification segment / GISTIC region in that sample. Consequently, the CN value is identical for all genes in a recurrent amplification / GISTIC region for a given sample. Spearman correlation was used to calculate the correlation between mRNA levels and partitioned CN log2ratios for each gene. In order for a gene to be included in the correlation analyses at least two samples with recurrent amplification, or at least 10 samples for GISTIC comparisons were required to have connected gene expression data. Notably, not all genes in recurrent amplifications / core GISTIC regions were present on all array types (U133A, U133 2plus, cDNA). Consequently, the number of cases used in the correlation analyses differs between genes. A Spearman correlation cut-off (0.18) representing P = 0.05 obtained from 10000 permutations of CN sample labels was used to identify significantly correlated genes in recurrent amplification regions. The corresponding Spearman cut-off (P = 0.05) for the core GISTIC regions was 0.22. Permutations were done separately for recurrent amplifications and core GISTIC regions, as number of genes differed in the two comparisons.

Analysis of differential mRNA expression for genes in recurrent amplifications between amplified samples (segmented CN >1) and samples without amplification was performed on a data set specific basis. At least four amplified samples with connected gene expression data was required for a gene to be tested in a data set. Wilcoxon’s test was used to identify differentially expressed genes between amplified and non-amplified samples, with a p-value threshold of 0.05 for identifying significant genes.
