# Supplementary material for: Landscape of somatic allelic imbalances and copy number alterations in HER2-amplified breast cancer
Source: Breast Cancer Res. 2011 Dec 14;13(6):R129. doi: 10.1186/bcr3075 (PMC3326571; doi:10.1186/bcr3075)
Supplement: Additional file 5 — CNAs in HER2-amplified breast tumors and cell lines. A pdf file containing six figures, S2A-F, showing the overlap of GISTIC regions identified in the current and a former BAC aCGH study, pattern of CNAs in 16 HER2-amplified breast cancer cell lines, shortest region of amplification including the HER2 gene, HER2-amplified cases defining the centromeric and telomeric breakpoints for the shortest region of amplification analysis, and GISTIC regions stratifying HER2-amplified breast cancer based on ER-status, respectively. [file bcr3075-S5.PDF]

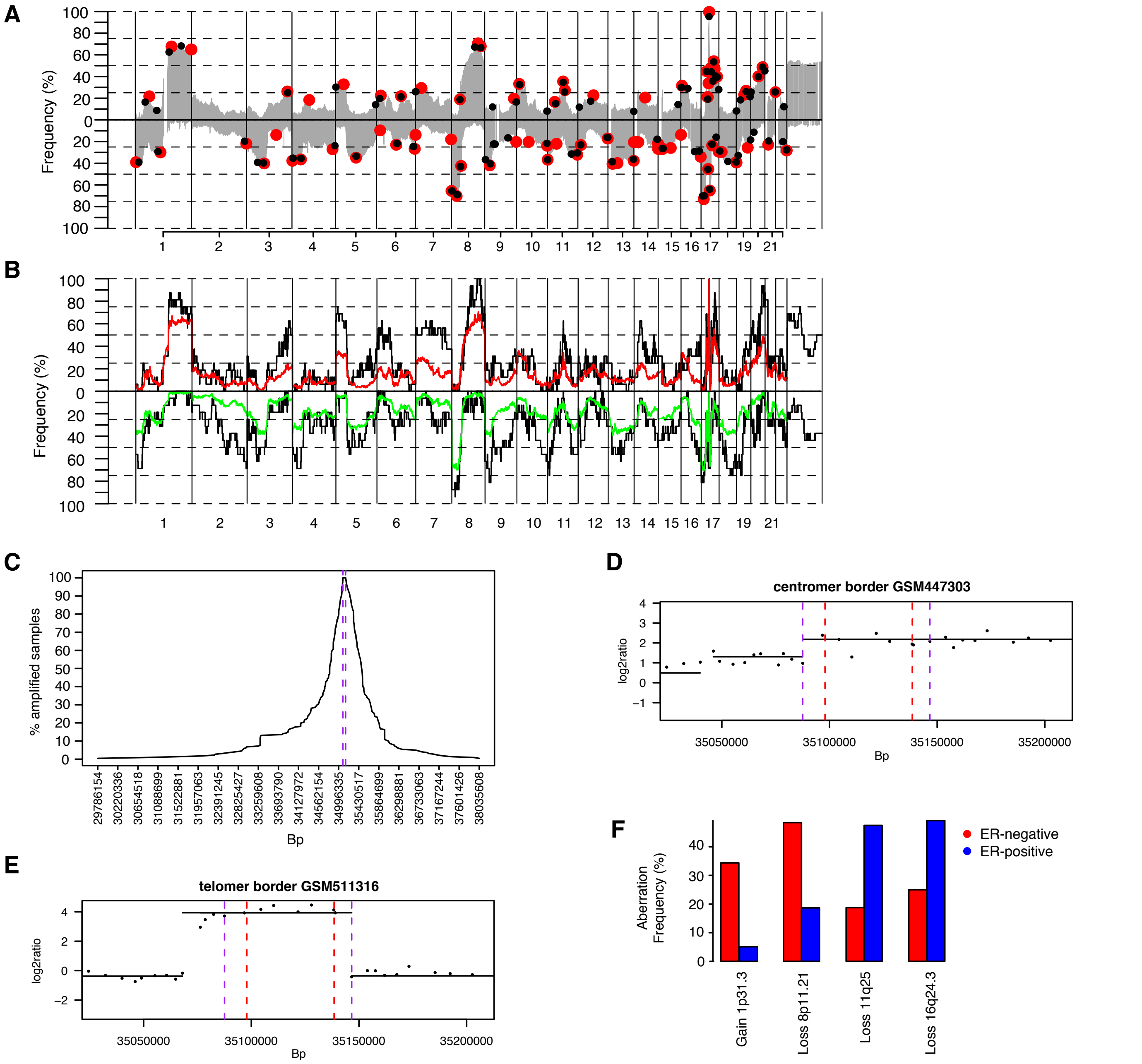

Supplementary Figure 2. Copy number alterations in HER2-amplified breast cancer. (**A**) Overlap of GISTIC regions in the current and a previous study comprising 200 HER2-amplified cases analyzed by 32K BAC aCGH [5]. Frequency of copy number gain and loss across the 218 HER2-amplified tumors in the current study is shown in grey. Red regions indicate GISTIC regions (n=80) identified from the 218 cases in the current study, while black regions (n=90) indicate GISTIC regions previously identified by BAC aCGH analysis of 200 HER2-amplified tumors [5] (remapped to hg18 coordinates and filtered for q-value < 0.05). Sizes of GISTIC regions are inflated for improved visualization. (**B**) Frequency of CN gain (black) and loss (black) across 16 HER2-amplified breast cancer cell lines analyzed by SNP microarrays. CN gain and loss are called using log2ratio thresholds of 0.12 (gain) and -0.12 (loss). The frequency of CN gain (red) and loss (green) obtained from GISTIC analysis of the 218 HER2-amplified primary tumors are shown for comparison. HER2-amplified cell lines include MDA-MB-361, MD-MBA-453_Hahn, UACC-893, UACC-812, SKBR3, SM-126O, AU5_65, EFM192A, HCC202, HCC1419, ZR7530, HCC1569, BT474, HCC2218, HCC1008, and HCC1954. (**C**) Shortest region of amplification including the complete *HER2* gene in the 218 tumors +16 cell lines. Y-axis shows percentage of cases with a specific amplicon size. Purple vertical line indicates shortest overlapping region chr17:35087594-35146597. The region was defined in a conservative manner using the start /stop assignments of the first / last probes not included in the HER2 segment plus/minus 1 bp. (**D**) Sample GSM447303 defining the centromeric limit. Purple line indicates the shortest region of amplification, red line the *HER2* gene (chr17: 35097919-35138441). Black lines correspond to partitioned segments. (**E**) Sample GSM511316 defining the telomeric limit. Purple line indicates the shortest region of amplification, red line the *HER2* gene. Black lines correspond to partitioned segments. (**F**) GISTIC regions stratifying HER2-amplified cases based on ER-status: ER-positive (n=59, blue), ER-negative (n=64, red). Regions identified by Fisher's test, with p-value < 0.01. GISTIC regions are defined in Additional file 3. Significant gain of GISTIC region 1p31.3 in ER-negative tumors in the current study is matched by a similar region of gain also on 1p31.3 in ER-negative tumors in our previous BAC-based aCGH study [5]. Significant loss of GISTIC region 16q24.3 in ER-positive tumors in the current study is matched by a similar region of loss on 16q23.3 in ER-positive tumors in our previous BAC-based aCGH study [5].
