# Supplementary material for: Landscape of somatic allelic imbalances and copy number alterations in HER2-amplified breast cancer
Source: Breast Cancer Res. 2011 Dec 14;13(6):R129. doi: 10.1186/bcr3075 (PMC3326571; doi:10.1186/bcr3075)
Supplement: Additional file 7 — Frequency of LOH and CNN-AI in HER2-negative breast cancers analyzed by GAP and stratified by PAM50 subtypes. A pdf file containing four figures, S3A-D showing frequency of LOH (upper panel) and CNN-AI (lower panel) for HER2-negative tumors classified as basal-like, luminal A, luminal B, and normal-like using PAM50. [file bcr3075-S7.PDF]

A)

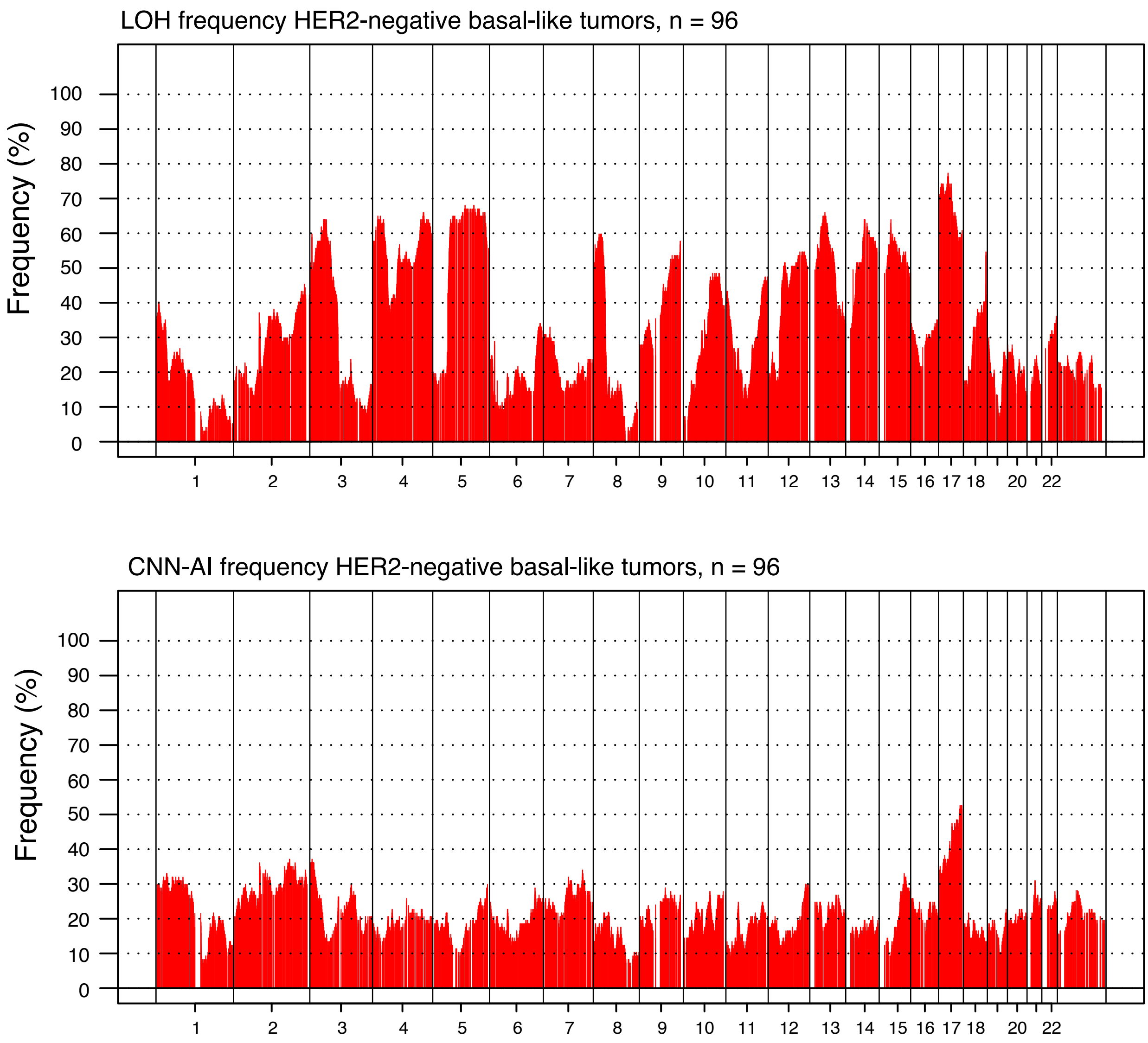

Supplementary Figure 3. Frequency of LOH and CNN-AI across GAP analysed HER2-negative breast cancers stratified by PAM50 molecular subtype. (**A**) Frequency of LOH (upper panel) and CNN-AI (lower panel) for 96 HER2-negative basal-like tumors. (**B**) Frequency of LOH (upper panel) and CNN-AI (lower panel) for 88 HER2-negative luminal A cases. (**C**) Frequency of LOH (upper panel) and CNN-AI (lower panel) for 90 HER2-negative luminal B cases. (**D**) Frequency of LOH (upper panel) and CNN-AI (lower panel) for 34 HER2-negative normal-like cases.

B)

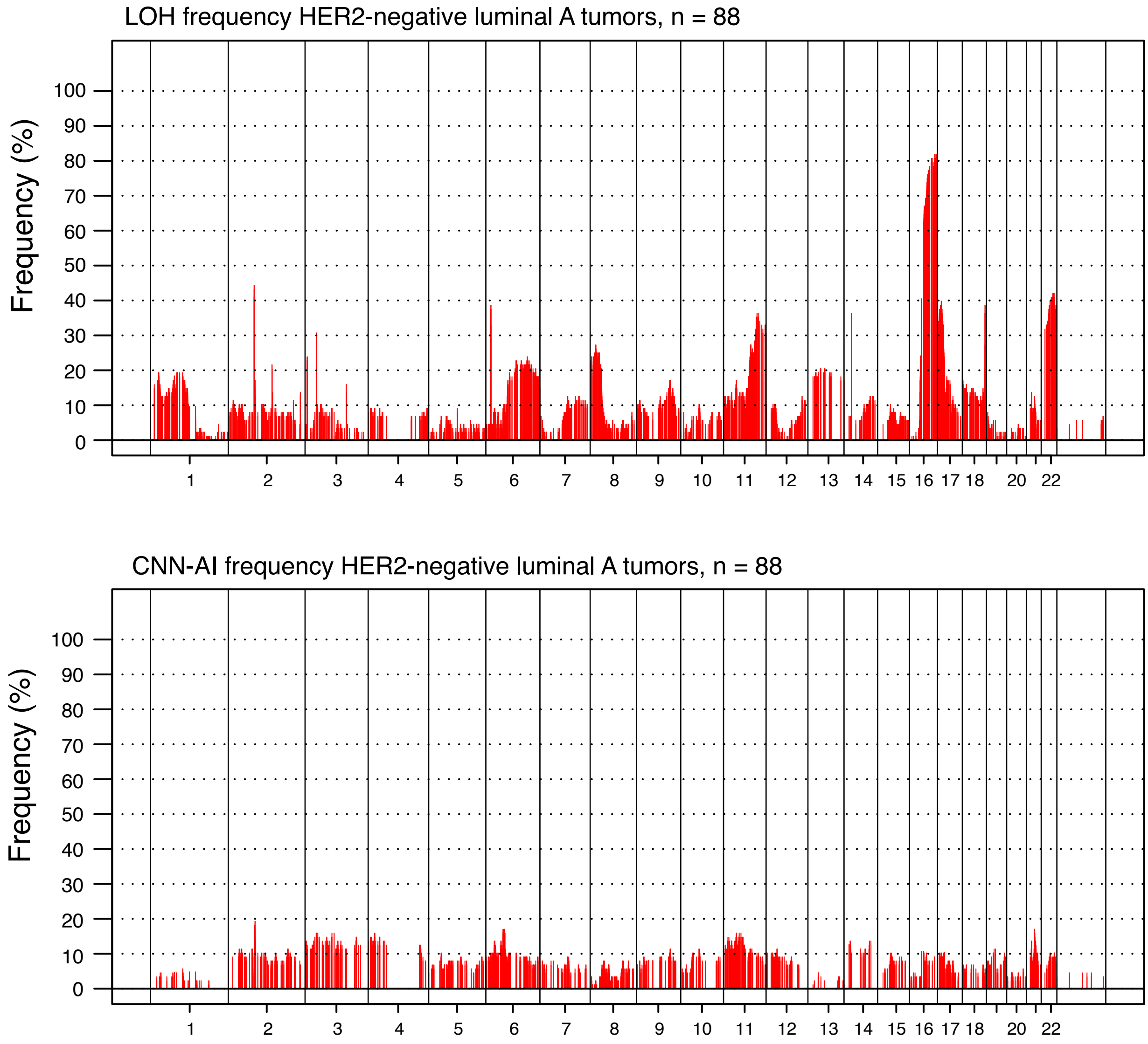
LOH frequency HER2-negative luminal A tumors, n = 88
Frequency (%)
0
10
20
30
40
50
60
70
80
90
100
1
2
3
4
5
6
7
8
9
10
11
12
13
14
15
16
17
18
20
22
CNN-AI frequency HER2-negative luminal A tumors, n = 88
Frequency (%)

C)

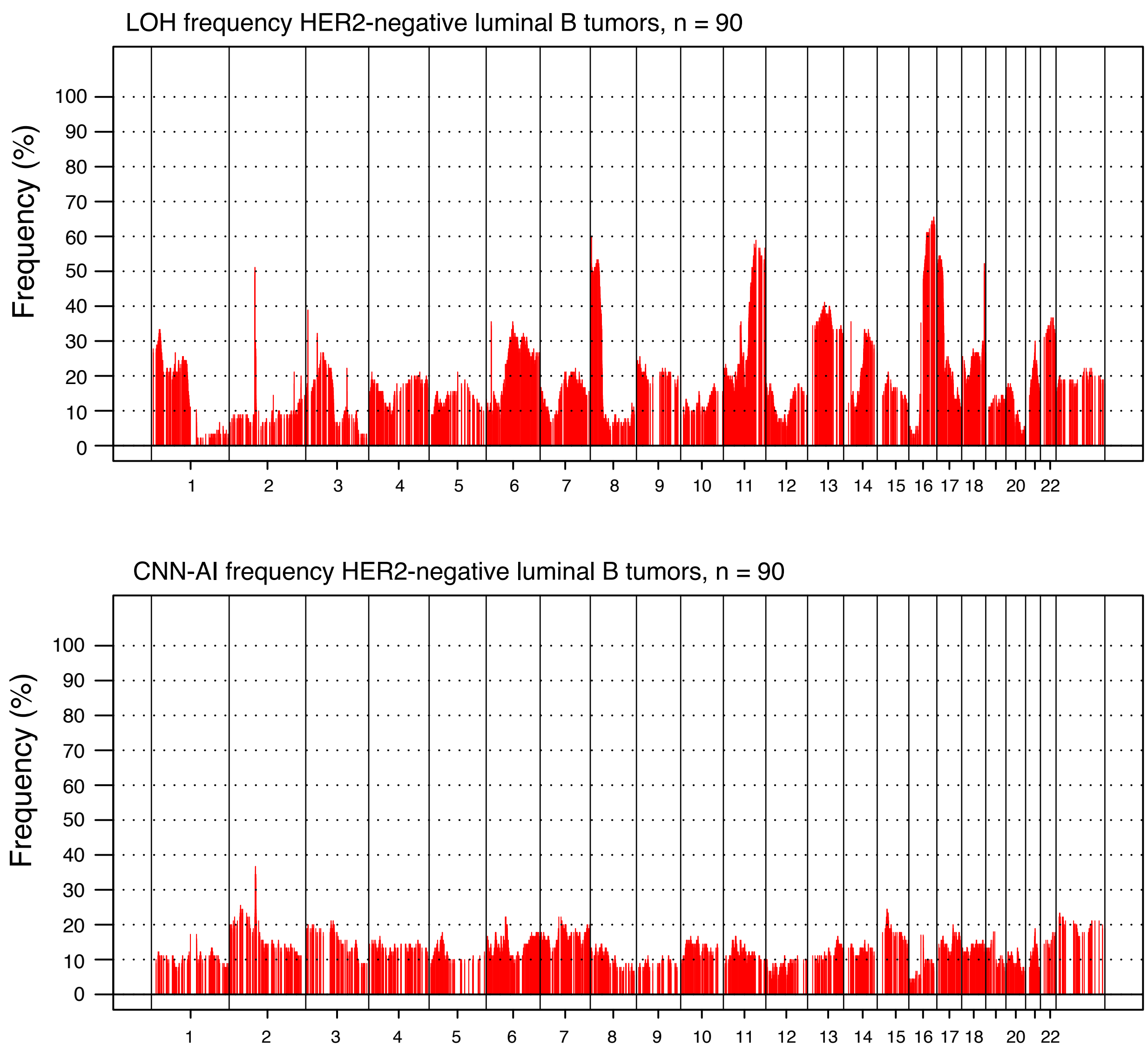

LOH frequency HER2-negative luminal B tumors, n = 90
Frequency (%)
100
90
80
70
60
50
40
30
20
10
0
1
2
3
4
5
6
7
8
9
10
11
12
13
14
15
16
17
18
20
22
CNN-AI frequency HER2-negative luminal B tumors, n = 90
Frequency (%)
100
90
80
70
60
50
40
30
20
10
0
1
2
3
4
5
6
7
8
9
10
11
12
13
14
15
16
17
18
20
22

D)

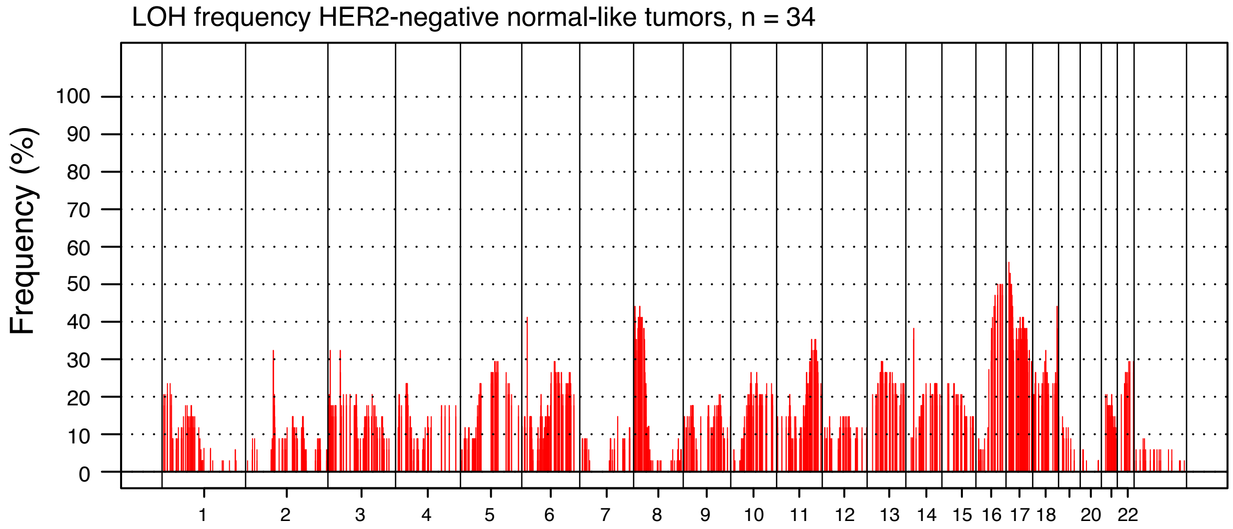

LOH frequency HER2-negative normal-like tumors, n = 34
Frequency (%)
100
90
80
70
60
50
40
30
20
10
0
1
2
3
4
5
6
7
8
9
10
11
12
13
14
15
16
17
18
20
22

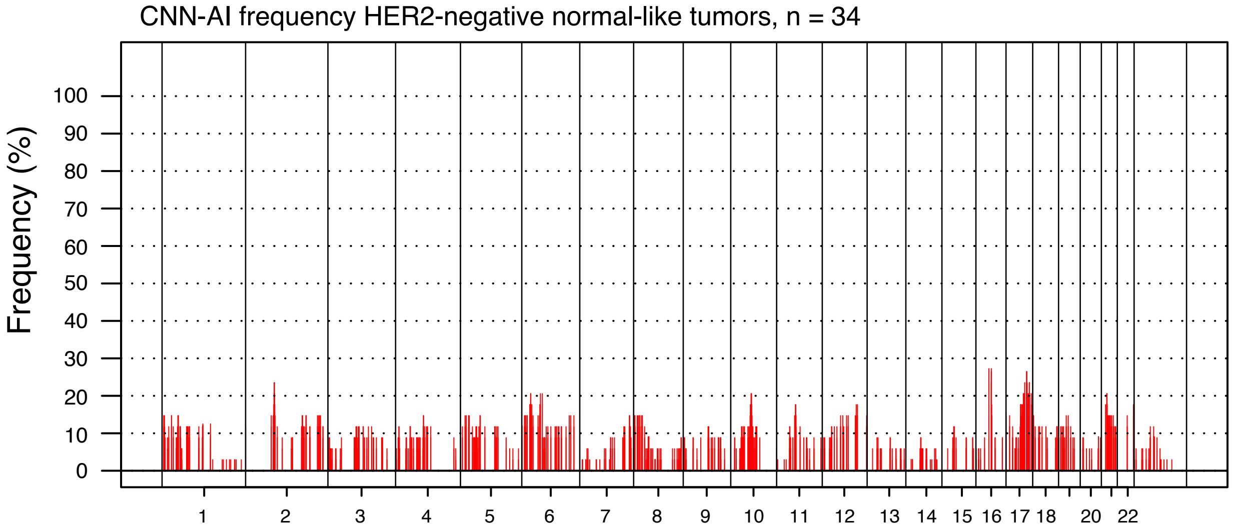

CNN-AI frequency HER2-negative normal-like tumors, n = 34
Frequency (%)
100
90
80
70
60
50
40
30
20
10
0
1
2
3
4
5
6
7
8
9
10
11
12
13
14
15
16
17
18
20
22
