# Supplementary material for: Landscape of somatic allelic imbalances and copy number alterations in HER2-amplified breast cancer
Source: Breast Cancer Res. 2011 Dec 14;13(6):R129. doi: 10.1186/bcr3075 (PMC3326571; doi:10.1186/bcr3075)
Supplement: Additional file 8 — Variation of FGA values versus GAP-ploidy for HER2-amplified and HER2-negative breast cancers. A pdf file containing five figures, S4A-E, showing the pattern of LOH-FGA, CNN-FGA and CN-FGA for HER2-amplified cases, HER2-negative basal-like tumors, HER2-negative luminal A tumors, HER2-negative luminal B tumors and HER2-negative normal-like tumors respectively. [file bcr3075-S8.PDF]

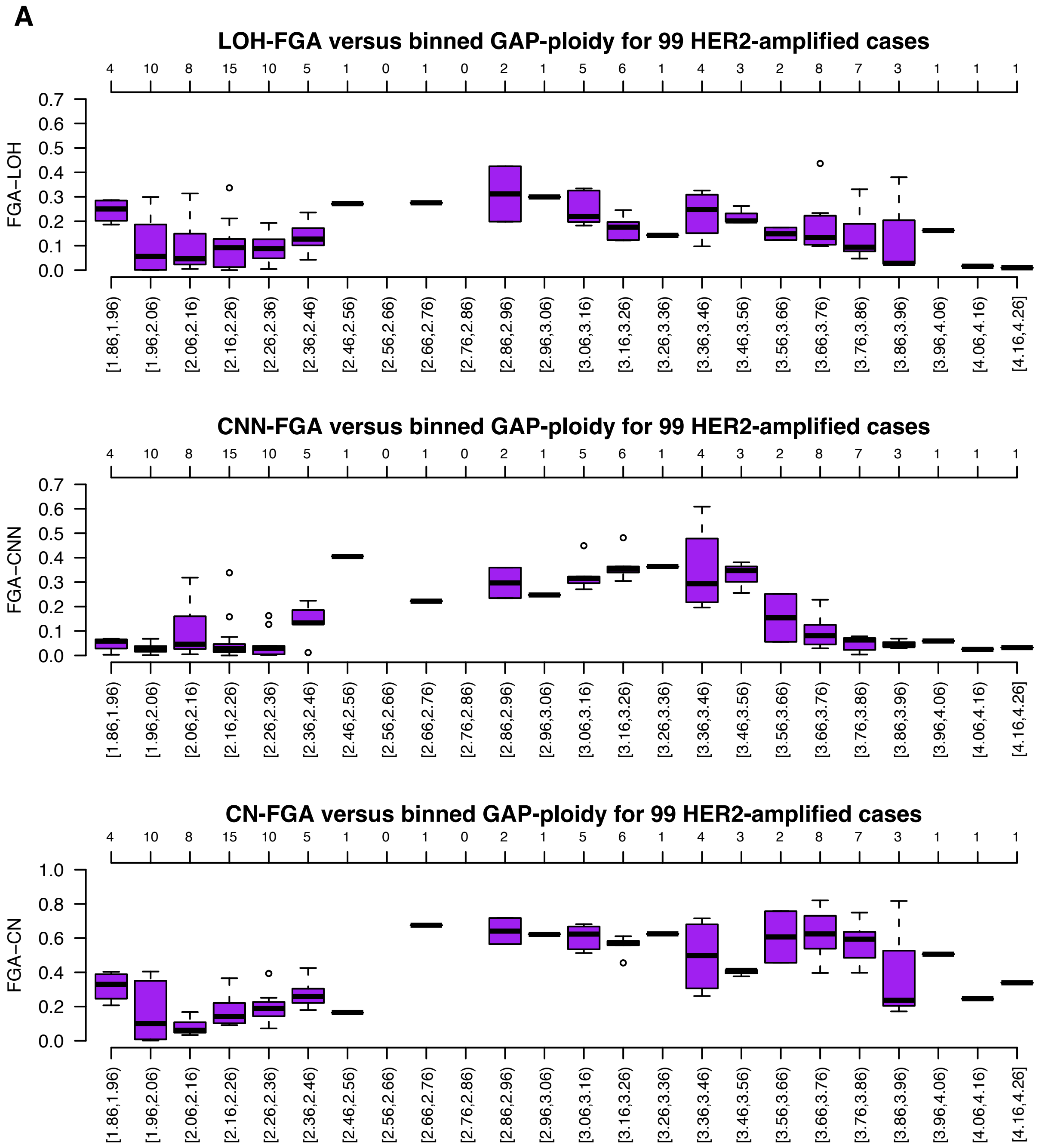

Supplementary Figure 4. Variation of FGA values versus GAP-ploidy for HER2-amplified and HER2-negative breast cancers. GAP-ploidy estimates were binned in 0.1 bins represented by tick marks on the x-axis. Bins contain different number of samples. For each bin a box plot is displayed for LOH-FGA (top), CNN-AI-FGA (center), and CN-FGA (bottom) for (**A**) 99 HER2-amplified cases, (**B**) 96 HER2-negative basal-like cases, (**C**) 88 HER2-negative luminal A cases, (**D**) 90 HER2-negative luminal B cases, and (**E**) 34 HER2-negative normal-like cases.

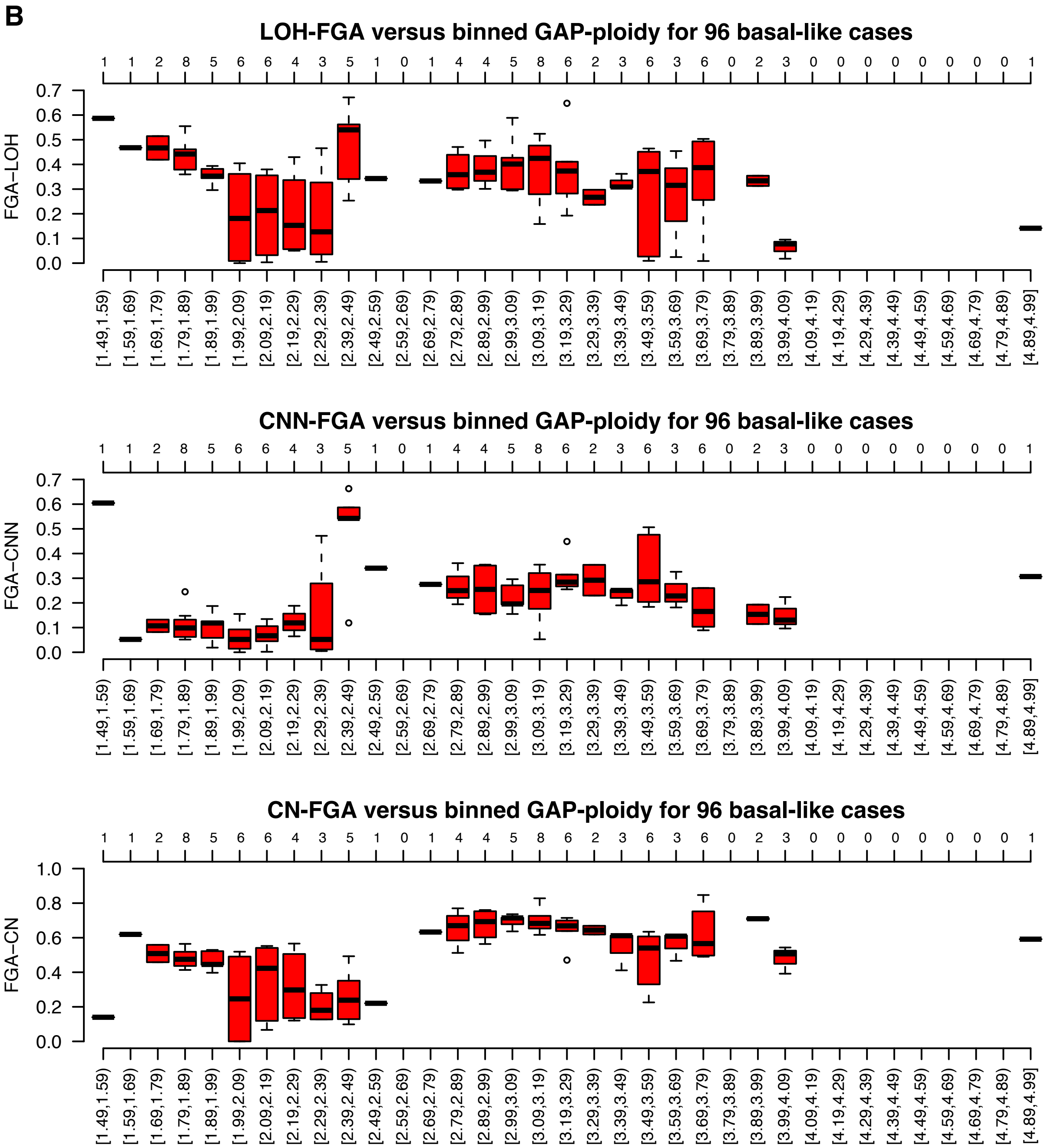
B
LOH-FGA versus binned GAP-ploidy for 96 basal-like cases
1 1 2 8 5 6 6 4 3 5 1 0 1 4 4 5 8 6 2 3 6 3 6 0 2 3 0 0 0 0 0 0 0 0 1
FGA−LOH
0.0 0.1 0.2 0.3 0.4 0.5 0.6 0.7
[1.49,1.59) [1.59,1.69) [1.69,1.79) [1.79,1.89) [1.89,1.99) [1.99,2.09) [2.09,2.19) [2.19,2.29) [2.29,2.39) [2.39,2.49) [2.49,2.59) [2.59,2.69) [2.69,2.79) [2.79,2.89) [2.89,2.99) [2.99,3.09) [3.09,3.19) [3.19,3.29) [3.29,3.39) [3.39,3.49) [3.49,3.59) [3.59,3.69) [3.69,3.79) [3.79,3.89) [3.89,3.99) [3.99,4.09) [4.09,4.19) [4.19,4.29) [4.29,4.39) [4.39,4.49) [4.49,4.59) [4.59,4.69) [4.69,4.79) [4.79,4.89) [4.89,4.99]
CNN-FGA versus binned GAP-ploidy for 96 basal-like cases
1 1 2 8 5 6 6 4 3 5 1 0 1 4 4 5 8 6 2 3 6 3 6 0 2 3 0 0 0 0 0 0 0 0 1
FGA−CNN
0.0 0.1 0.2 0.3 0.4 0.5 0.6 0.7
[1.49,1.59) [1.59,1.69) [1.69,1.79) [1.79,1.89) [1.89,1.99) [1.99,2.09) [2.09,2.19) [2.19,2.29) [2.29,2.39) [2.39,2.49) [2.49,2.59) [2.59,2.69) [2.69,2.79) [2.79,2.89) [2.89,2.99) [2.99,3.09) [3.09,3.19) [3.19,3.29) [3.29,3.39) [3.39,3.49) [3.49,3.59) [3.59,3.69) [3.69,3.79) [3.79,3.89) [3.89,3.99) [3.99,4.09) [4.09,4.19) [4.19,4.29) [4.29,4.39) [4.39,4.49) [4.49,4.59) [4.59,4.69) [4.69,4.79) [4.79,4.89) [4.89,4.99]
CN-FGA versus binned GAP-ploidy for 96 basal-like cases
1 1 2 8 5 6 6 4 3 5 1 0 1 4 4 5 8 6 2 3 6 3 6 0 2 3 0 0 0 0 0 0 0 0 1
FGA−CN
0.0 0.2 0.4 0.6 0.8 1.0
[1.49,1.59) [1.59,1.69) [1.69,1.79) [1.79,1.89) [1.89,1.99) [1.99,2.09) [2.09,2.19) [2.19,2.29) [2.29,2.39) [2.39,2.49) [2.49,2.59) [2.59,2.69) [2.69,2.79) [2.79,2.89) [2.89,2.99) [2.99,3.09) [3.09,3.19) [3.19,3.29) [3.29,3.39) [3.39,3.49) [3.49,3.59) [3.59,3.69) [3.69,3.79) [3.79,3.89) [3.89,3.99) [3.99,4.09) [4.09,4.19) [4.19,4.29) [4.29,4.39) [4.39,4.49) [4.49,4.59) [4.59,4.69) [4.69,4.79) [4.79,4.89) [4.89,4.99]

C

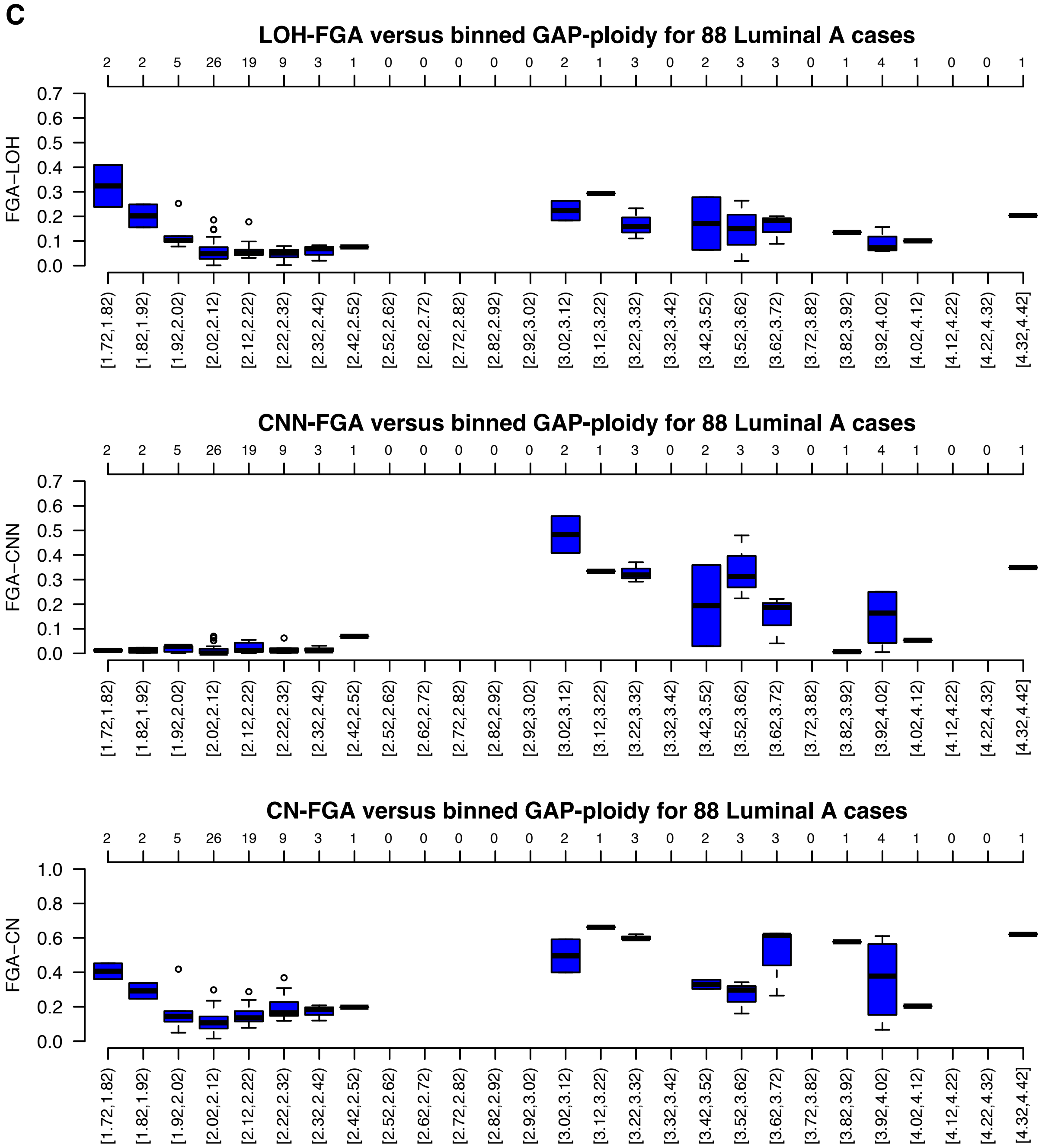

LOH-FGA versus binned GAP-ploidy for 88 Luminal A cases
2 2 5 26 19 9 3 1 0 0 0 0 0 2 1 3 0 2 3 3 0 1 4 1 0 0 1
FGA−LOH
[1.72,1.82) [1.82,1.92) [1.92,2.02) [2.02,2.12) [2.12,2.22) [2.22,2.32) [2.32,2.42) [2.42,2.52) [2.52,2.62) [2.62,2.72) [2.72,2.82) [2.82,2.92) [2.92,3.02) [3.02,3.12) [3.12,3.22) [3.22,3.32) [3.32,3.42) [3.42,3.52) [3.52,3.62) [3.62,3.72) [3.72,3.82) [3.82,3.92) [3.92,4.02) [4.02,4.12) [4.12,4.22) [4.22,4.32) [4.32,4.42]
CNN-FGA versus binned GAP-ploidy for 88 Luminal A cases
2 2 5 26 19 9 3 1 0 0 0 0 0 2 1 3 0 2 3 3 0 1 4 1 0 0 1
FGA−CNN
CN-FGA versus binned GAP-ploidy for 88 Luminal A cases
2 2 5 26 19 9 3 1 0 0 0 0 0 2 1 3 0 2 3 3 0 1 4 1 0 0 1
FGA−CN

D

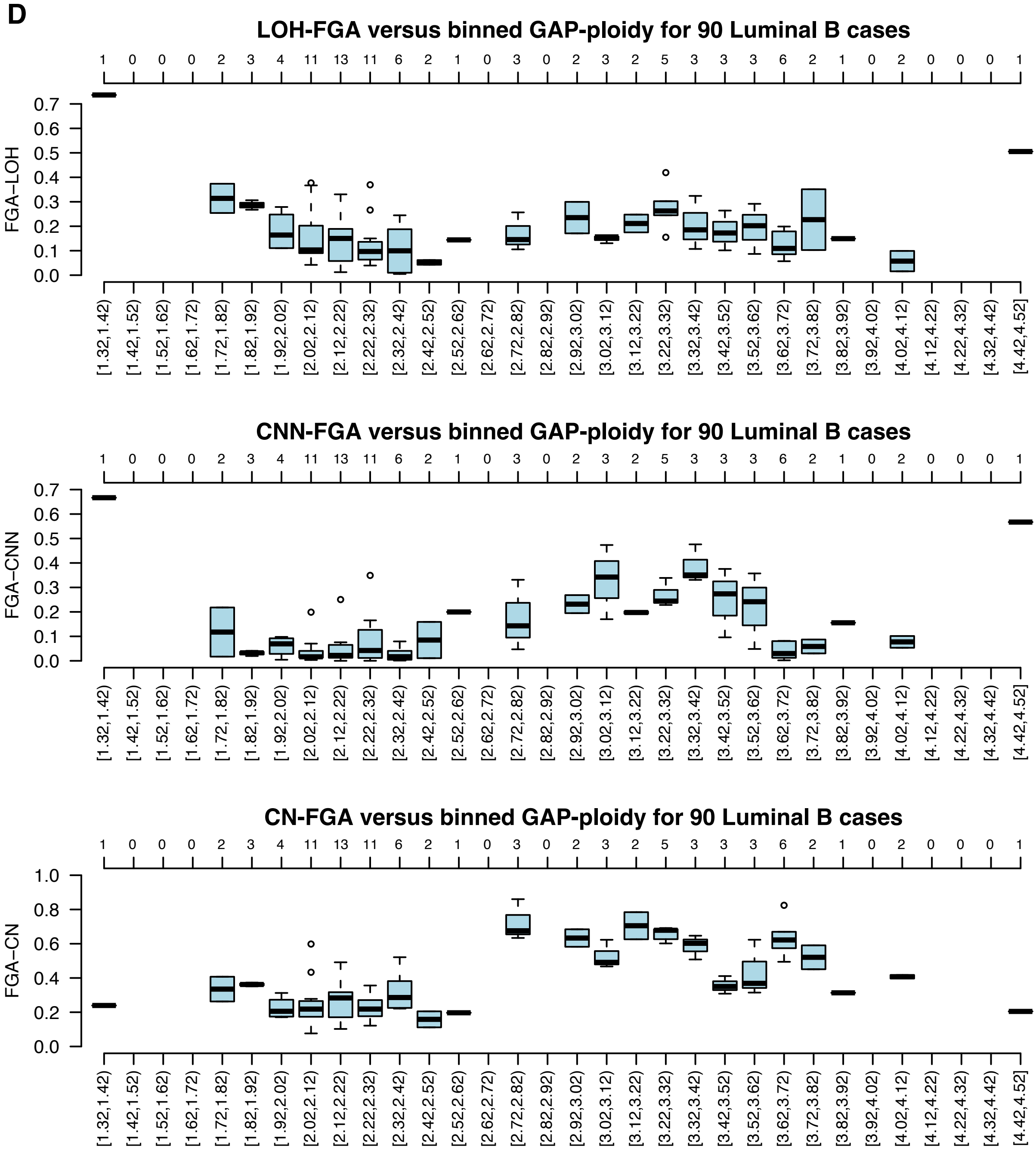
LOH-FGA versus binned GAP-ploidy for 90 Luminal B cases
FGA–LOH
CNN-FGA versus binned GAP-ploidy for 90 Luminal B cases
FGA–CNN
CN-FGA versus binned GAP-ploidy for 90 Luminal B cases
FGA–CN
1 0 0 0 2 3 4 11 13 11 6 2 1 0 3 0 2 3 2 5 3 3 3 6 2 1 0 2 0 0 0 1
[1.32,1.42) [1.42,1.52) [1.52,1.62) [1.62,1.72) [1.72,1.82) [1.82,1.92) [1.92,2.02) [2.02,2.12) [2.12,2.22) [2.22,2.32) [2.32,2.42) [2.42,2.52) [2.52,2.62) [2.62,2.72) [2.72,2.82) [2.82,2.92) [2.92,3.02) [3.02,3.12) [3.12,3.22) [3.22,3.32) [3.32,3.42) [3.42,3.52) [3.52,3.62) [3.62,3.72) [3.72,3.82) [3.82,3.92) [3.92,4.02) [4.02,4.12) [4.12,4.22) [4.22,4.32) [4.32,4.42) [4.42,4.52]

E

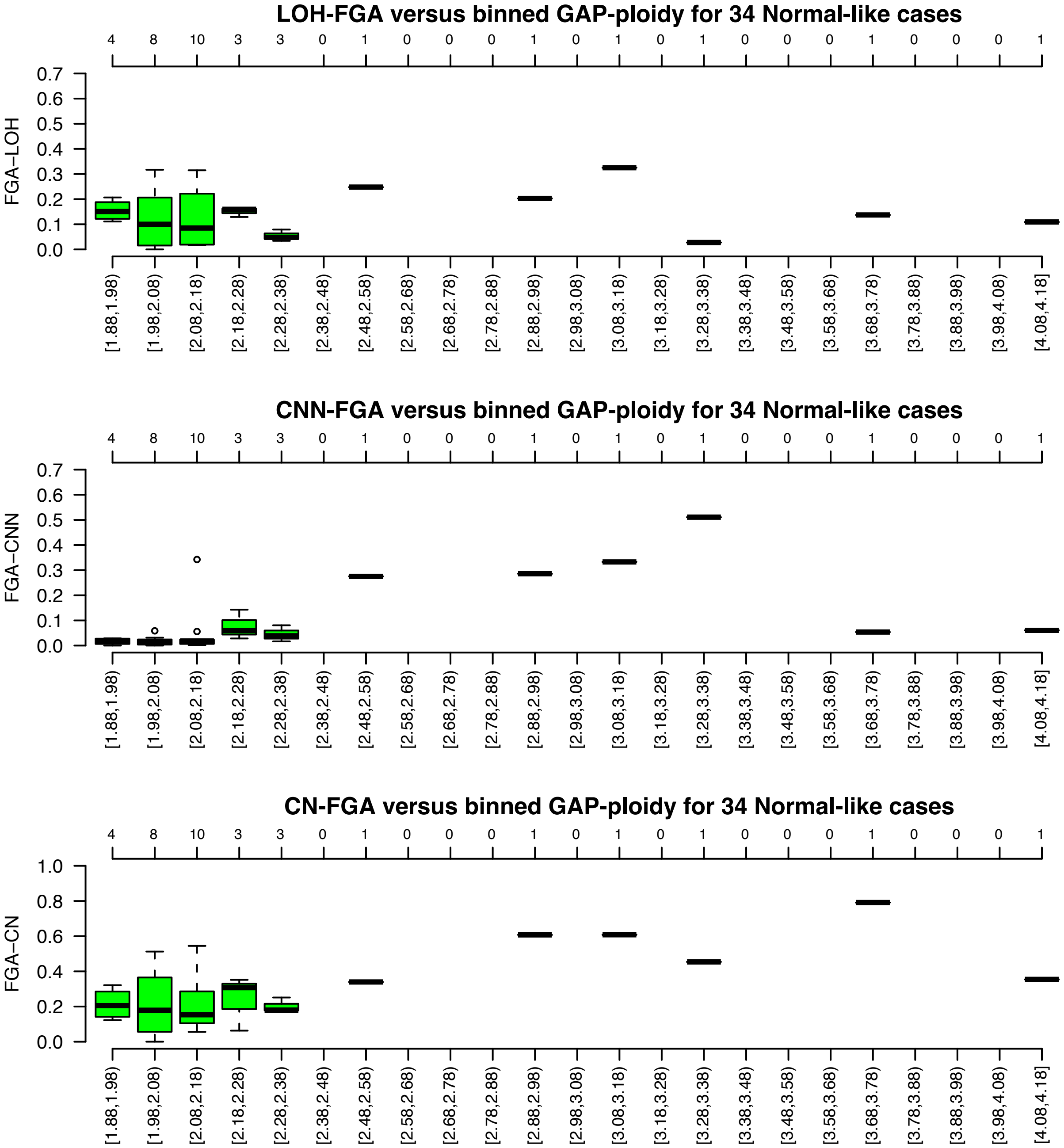
LOH-FGA versus binned GAP-ploidy for 34 Normal-like cases
FGA−LOH
CNN-FGA versus binned GAP-ploidy for 34 Normal-like cases
FGA−CNN
CN-FGA versus binned GAP-ploidy for 34 Normal-like cases
FGA−CN
4 8 10 3 3 0 1 0 0 0 1 0 1 0 1 0 0 0 1 0 0 0 1
[1.88,1.98) [1.98,2.08) [2.08,2.18) [2.18,2.28) [2.28,2.38) [2.38,2.48) [2.48,2.58) [2.58,2.68) [2.68,2.78) [2.78,2.88) [2.88,2.98) [2.98,3.08) [3.08,3.18) [3.18,3.28) [3.28,3.38) [3.38,3.48) [3.48,3.58) [3.58,3.68) [3.68,3.78) [3.78,3.88) [3.88,3.98) [3.98,4.08) [4.08,4.18]
