# Supplementary material for: Landscape of somatic allelic imbalances and copy number alterations in HER2-amplified breast cancer
Source: Breast Cancer Res. 2011 Dec 14;13(6):R129. doi: 10.1186/bcr3075 (PMC3326571; doi:10.1186/bcr3075)
Supplement: Additional file 9 — Tumor ploidy for HER2-amplified cases estimated by GAP analysis. A pdf file containing two figures, S5A-B, showing the distribution of GAP-ploidy estimates for 407 HER2-amplified and HER2-negative cases stratified according to subtype, and HER2-amplified cases stratified by ER-status respectively. [file bcr3075-S9.PDF]

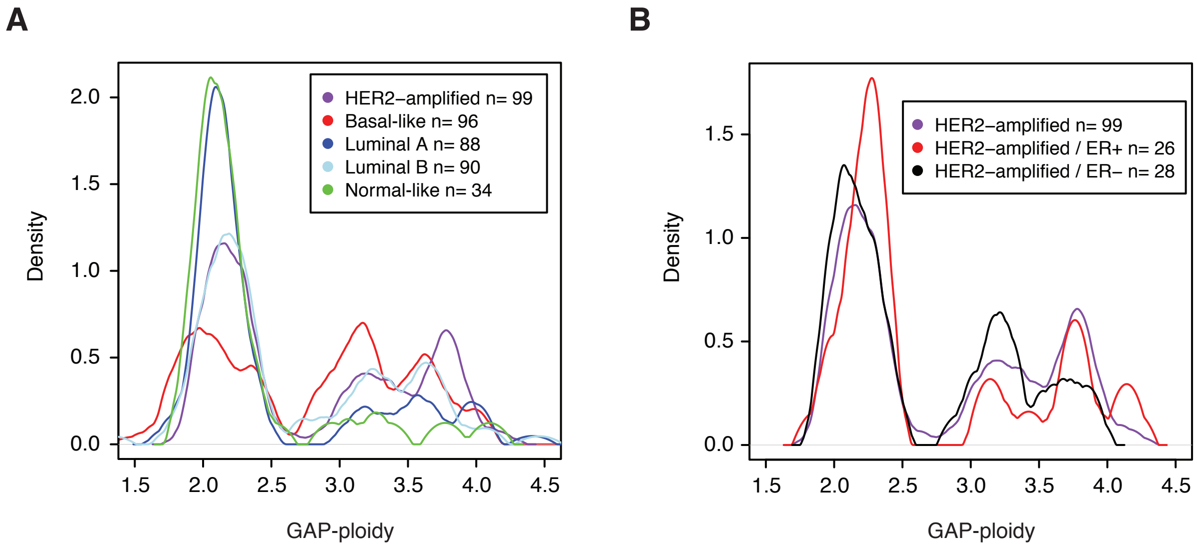

Supplementary Figure 5. Tumor ploidy for HER2-amplified cases estimated by GAP analysis. (**A**) Distribution of GAP-ploidy estimates across 407 breast cancer samples grouped as HER2-amplified by SNP array analysis, or HER2-negative by SNP array analysis and further stratified by PAM50 subtype. (**B**) Distribution of GAP-ploidy estimates for 54 HER2-amplified cases analyzed by SNP arrays and stratified by ER-status. In A and B, curves were generated by an Epanechnikov smoothing kernel with 0.08 smoothing bandwidth.
